# Modulating the DNA/Lipid Interface through Multivalent Hydrophobicity

**DOI:** 10.1021/acs.nanolett.4c02564

**Published:** 2024-07-26

**Authors:** Siu Ho Wong, Sarina Nicole Kopf, Vincenzo Caroprese, Yann Zosso, Diana Morzy, Maartje M. C. Bastings

**Affiliations:** † Programmable Biomaterials Laboratory, Institute of Materials, School of Engineering, Ecole Polytechnique Fédérale Lausanne, Lausanne 1015, Switzerland; ‡ Interfaculty Bioengineering Institute, School of Engineering, Ecole Polytechnique Fédérale Lausanne, Lausanne 1015, Switzerland

**Keywords:** hydrophobic anchors, DNA-membrane interactions, multivalency, DNA nanotechnology

## Abstract

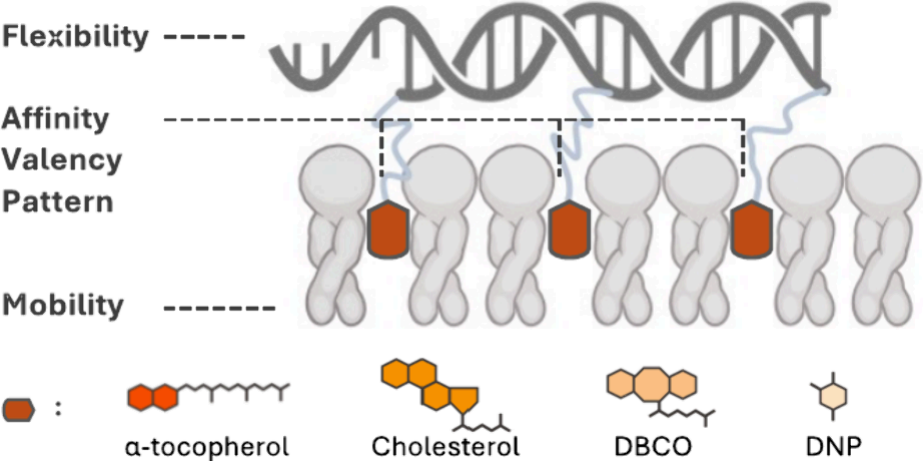

Lipids and nucleic acids are two of the most abundant components of our cells, and both molecules are widely used as engineering materials for nanoparticles. Here, we present a systematic study of how hydrophobic modifications can be employed to modulate the DNA/lipid interface. Using a series of DNA anchors with increasing hydrophobicity, we quantified the capacity to immobilize double-stranded (ds) DNA to lipid membranes in the liquid phase. Contrary to electrostatic effects, hydrophobic anchors are shown to be phase-independent if sufficiently hydrophobic. For weak anchors, the overall hydrophobicity can be enhanced following the concept of multivalency. Finally, we demonstrate that structural flexibility and anchor orientation overrule the effect of multivalency, emphasizing the need for careful scaffold design if strong interfaces are desired. Together, our findings guide the design of tailored DNA/membrane interfaces, laying the groundwork for advancements in biomaterials, drug delivery vehicles, and synthetic membrane mimics for biomedical research and nanomedicine.

Nucleic acids (NA) and lipids are essential constituents of all cells yet are also key components of many synthetically engineered nanostructures, including liposomes, vaccines, and DNA origami nanoparticles. The latter are often used to interact with our cells, creating a reciprocal interplay of natural and synthetic arrangements among the same core molecules. This dual system creates a series of artificial interfaces which remain largely underexplored yet are highly decisive for a robust and predictive performance of biomaterials. Therefore, understanding and manipulating this interplay between NA and lipids are relevant for the engineering of new tools toward biomedical applications.

Formulations integrating NA and lipids are indispensable in facilitating cell transfection^1,2^ and gene therapy,^3,4^ with NA vaccines serving as a prime example with clear medical and societal impact.^4−7^ Analysis of NA–lipid interactions has primarily focused on electrostatic binding, occurring between the negative charge of the phosphate backbone and the varied charge profiles of lipid headgroups.^8^ Cationic or ionizable lipids have been incorporated into NA formulations to enhance electrostatic interactions,^5,9^ abundantly used in NA vaccine development. Moreover, the membrane mobility (e.g., liquid or gel phase) as well as the structural properties of DNA have been shown to impact the electrostatic binding between these molecules. Besides electrostatic bridging, DNA can be chemically modified with hydrophobic moieties that serve as anchors to facilitate stronger attachment to the lipid membrane.^10^ This synthetic hydrophobic modification has broadened the functional capabilities of programmable DNA nanostructures thanks to ease of functionalization, simplicity, and robustness.^11^ Examples include the formation of synthetic membrane channels and insertion of nanopores for molecular transport across membranes.^12,13^ Moreover, membranes can be equipped with “artificial receptors”, which are sequence-controlled cholesterol–DNA conjugates that allow engineering of functional interfaces through programmed interactions.^14,15^

Research on hydrophobically modified DNA nanostructures has primarily focused on cholesterol-mediated interactions with model membranes.^16,17^ Strategies for enhancing binding include neutralizing membrane charges with divalent ions,^10,18^ increasing anchor numbers,^19,20^ positioning of terminal anchors,^21^ and enhancing molecular accessibility through flexible spacer chains like tetraethylene glycol (TEG).^16,19^ However, a systematic study of more diverse hydrophobic interactions between DNA and membranes remains elusive. Fundamental questions arise regarding the variations in hydrophobicity of molecules affecting binding affinities and, furthermore, the effects of changes in their number and position on the interactions with lipids.

In this study, we aimed to quantify how anchor hydrophobicity, valency, spatial orientation, and DNA structural design interplay toward the robust interaction with lipid membranes in the liquid and gel phases. Combining confocal imaging with zeta potential measurements, we investigate a range of molecules with varying hydrophobicity and analyze their impact on membrane attachment. We find that a multivalency effect depends on the anchor’s intrinsic hydrophobicity and significantly affects anchors with moderate-to-weak affinity. Furthermore, we explore the contributions of DNA rigidity and anchor patterns to the attachment strength, showing that structural flexibility negatively affects binding within structures of equal valency. This systematic investigation allows us to widen the hydrophobicity toolbox to engineer robust DNA/membrane interfaces and propose methods to fine-tune the stability of hydrophobically modified nanostructures.

Interactions between DNA and zwitterionic lipid membranes can occur via electrostatic bridging, where divalent cations “bridge” between both the negatively charged phosphates on the DNA backbone and zwitterionic lipids.^18^ This results in the attachment of DNA to lipid membranes, though we previously demonstrated this only happens when both DNA and the membrane are structurally rigid or if many interactions can be made, e.g., for large nucleic acid architectures.^22^ Using confocal microscopy, the interaction between DNA and phosphatidylcholine (PC) membranes in the form of giant unilamellar vesicles (GUVs) can be visualized. With a reduced entropic penalty of binding, dsDNA and gel-phase lipid membranes readily interact in the presence of divalent cations, but no interaction is present on the more mobile liquid-phase surfaces (Figure 1a). A more robust attachment of DNA to lipid membranes can be achieved when hydrophobic moieties such as cholesterol are connected to the DNA (Figure S1a, Table S1), inducing a hydrophobic interaction with the lipid tails. Comparing the difference between electrostatic-mediated and hydrophobic-mediated binding, we directly observed that the cholesterol anchors function independently of the lipid phase (Figure 1b).

**Figure 1:**
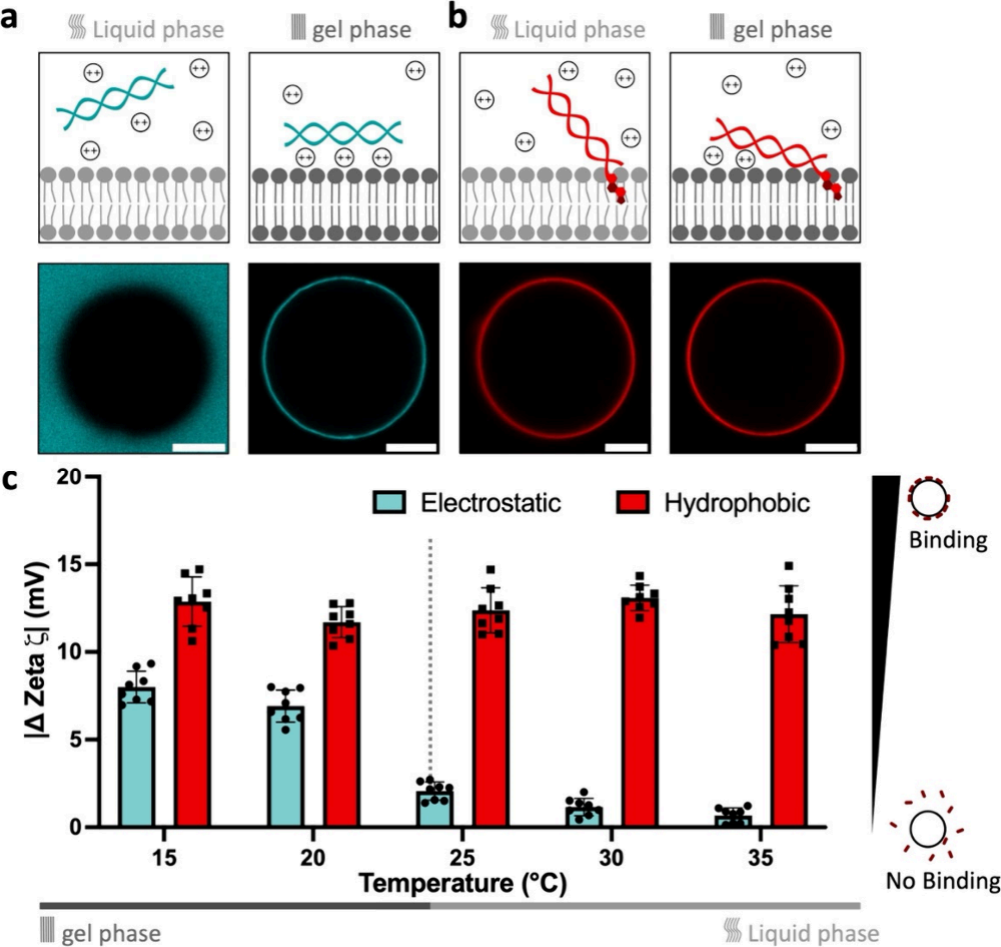
Electrostatic bridging versus hydrophobic anchoring between DNA and zwitterionic lipid bilayers. (a) Schematic representation (top) of electrostatic-driven interactions which solely occur in the gel phase, as shown by confocal micrographs of Cy5-labeled dsDNA incubated with POPC (liquid phase) or DPPC (gel phase) GUVs at room temperature. Contrarily, (b) hydrophobic-mediated interactions, here driven by a single cholesterol, occur in both phases. (c) Zeta potential measurements showing robust lipid phase-independent attachment of negatively charged dsDNA to DMPC LUVs across a wide range of temperatures, whereas electrostatic interactions are measured only at low temperature (i.e., gel phase). The error bar represents standard deviation from eight replicates, each consisting of three measurements, each with at least 15 subruns. Dashed line indicating the transition temperature of DMPC lipids at 24 °C. Scale bar: 10 μm.

Curious about how the lipid phase could gradually tune DNA attachment, electrophoretic light scattering (ELS) was applied. Since this technique operates on smaller particles, we generated large unilamellar vesicles (LUVs, 115.73 ± 5.1 nm, Figure S2) from zwitterionic DMPC (transition temperature *T*_m_ = 24 °C) and incubated these with unmodified and cholesterol-modified dsDNA (Figure S1b) in a range of temperatures below and above the lipid *T*_m_. We measured the absolute change in ζ potential (|Δζ| = |ζ_LUV+DNA_ – ζ_LUV_|) of the vesicles after 5 min of incubation with the DNA duplexes. Starting at a low temperature, both hydrophobic and electrostatic interactions occur. However, when gradually heating and crossing the *T*_m_, we observe a direct drop for electrostatic binding, while the cholesterol-mediated interactions are not altered at all (Figure 1c). The observed behavior across both techniques suggests that the hydrophobic attachment is independent of the bilayer’s mobility and diffusivity. We note that the different PC lipids used in these measurements contain identical zwitterionic headgroups to not affect the DNA–lipid interface. This allows us to adequately screen the effects of the lipids’ phase and mobility over a range of temperatures (Table S2).

While the robust interaction of cholesterol comes as no surprise, this molecule is less suited when a more dynamic range of DNA–lipid interactions is desired. In a similar fashion, other molecules can be used as hydrophobic anchors, and we narrowed our analysis to a small set of hydrophobic moieties available for DNA conjugation (Figure 2a). Each of these is connected to the 5-prime of DNA with a tetraethylene glycol (TEG) as a spacer (Figure S3a), facilitating crossing the polar head groups before settling into the hydrophobic inner membrane domain. α-Tocopherol (α-toco) and cholesterol (Chol) have been demonstrated to incorporate into lipid membranes for targeting lipid domains^23^ and phase-partitioning of DNA nanostructures,^24^ while dibenzocyclooctyne (DBCO) and 2,4-dinitrophenol (DNP) remain unexplored. We categorized the hydrophobicity of these anchors based on their partition coefficients (log *P*),^25^ which represent the distribution of the compound between the hydrophobic and hydrophilic phases (i.e., polar or nonpolar solvents; Figure 2b).

**Figure 2:**
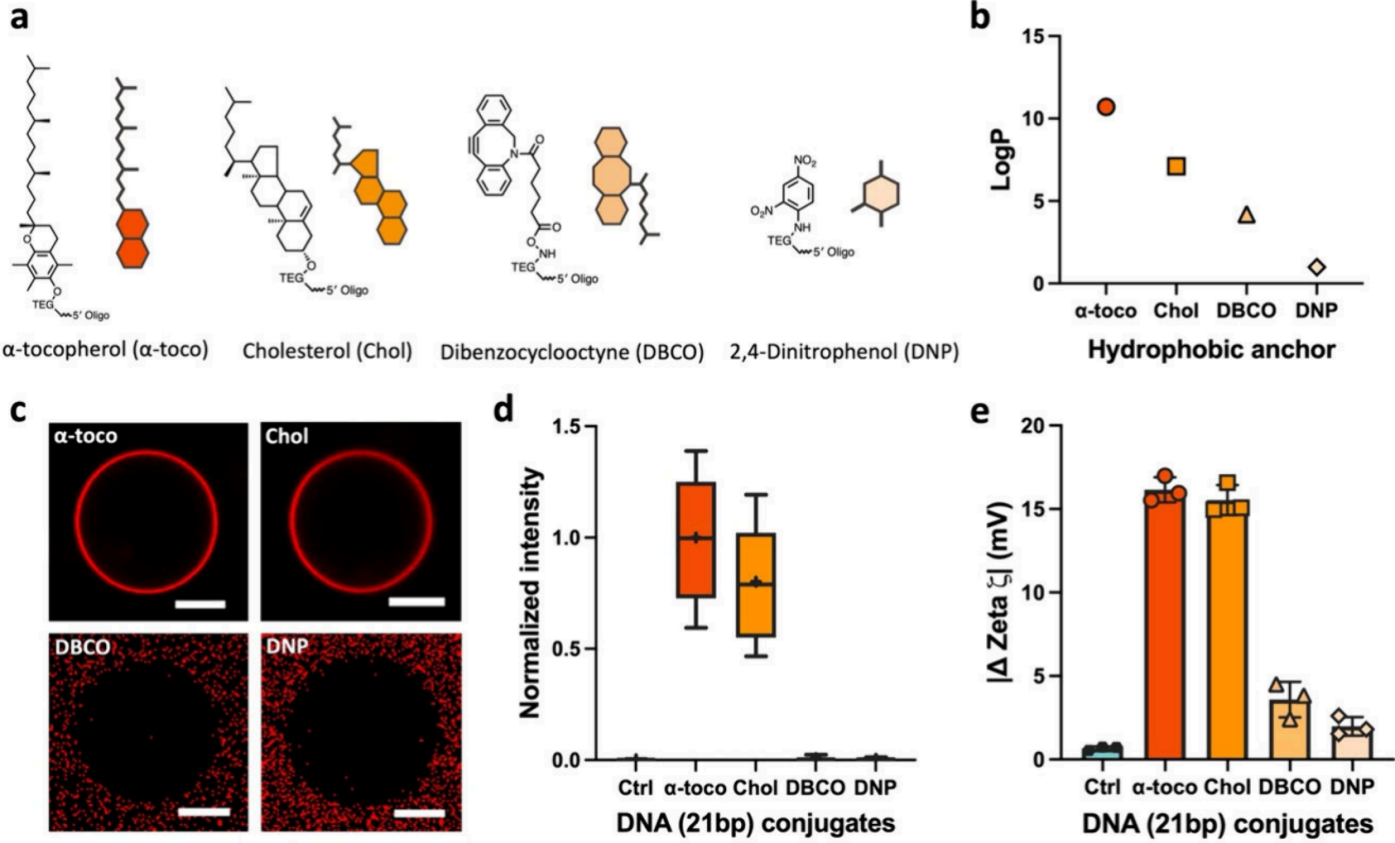
Varying the hydrophobicity of the modified anchors dictates the affinity of modified DNA to PC lipid membranes. (a) Overview of the chemical structures and cartoon representation of the various hydrophobic anchors used in this study. (b) The hydrophobicity variation present in the anchor ensemble, represented by the partition coefficient (log *P*). (c) Representative micrographs presenting a qualitative comparison of the binding affinity of various hydrophobically anchored DNA. Scale bar: 10 μm. (d) Qualitative analysis of modified DNA attachment to POPC GUV (liquid phase) at 37 °C, measured as fluorescent signals around vesicles. Box plots represent values measured from three replicates, each with 10 images containing at least 180 vesicles in total. (e) ζ potential measurement of anchored DNA attachment to DMPC LUV, where the error bar represents standard deviation from three replicates, each consisting of three measurements, each with at least 15 subruns.

We visualized the interaction of these hydrophobic DNA conjugates (Figure S3b) with freshly prepared POPC GUVs in the liquid phase, to eliminate the electrostatic component to DNA/lipid binding (Figure 2c). Both α-toco- and Chol-modified DNA showed strong lipid binding, but rarely any interactions were measured for the less hydrophobic DBCO and DNP conjugates. This observation suggests that a certain hydrophobicity is required to enable robust membrane anchoring. As the laser settings in the Cy5 channel were standardized across samples, we quantified the binding across anchor species by integration of the intensity (Figure 2d). Unmodified (UM) 21bp DNA was included as a negative control for electrostatic background interaction, which shows a minimal signal.

To further strengthen our findings that the hydrophobicity of modified anchors directly correlates with the binding of DNA to lipid membranes, ζ potential measurements were performed. We incubated these anchor-modified DNA (Figure S3) with freshly prepared DMPC LUVs in the liquid phase (118.73 ± 10.19 nm, Figure S2b). The results presented in Figure 2e demonstrate the effect of the various modified anchors with a-toco and Chol presenting a strong interaction. Again, we observed minimal interaction for DBCO and DNP anchors, confirming that a certain hydrophobicity is required for stable membrane anchoring. Moreover, we measured the same trends using single stranded DNA (ssDNA) conjugates (Figure S4a,b), indicating that binding seems dominated by hydrophobicity. Hydrophobicity as an inherent property thus directly offers simple tunability and provides simple options to fine-tune interactions between DNA-based biomaterials and lipid membranes (Figure S4c).

The observed differences in hydrophobicity-guided membrane binding between strong and weak anchors suggest parallels to high- and low-affinity ligand–receptor interactions. As such, the strength in numbers concept of multivalency^26−28^ could be employed to transform our weak anchors into strong ensembles. To combine multiple anchors, we extended our DNA duplex to 84 bp enabling hybridization of up to four anchors (Figure 3a, Figure S5). The change in ζ potential was measured (Figure 3b) and showed a minimal effect for the strongly hydrophobic anchors, yet a significant multivalency effect was observed for the weaker DBCO anchor. DNP, being the weakest of the group, could not benefit from multimerization, suggesting its overall hydrophobicity was still insufficient. Of note, a clear difference between one- and two-cholesterol anchors was present, consistent with literature reports stating a single cholesterol association is relatively moderate,^10^ whereas a combination of two cholesterols almost invariably led to irreversible attachment to membranes.^23^

**Figure 3:**
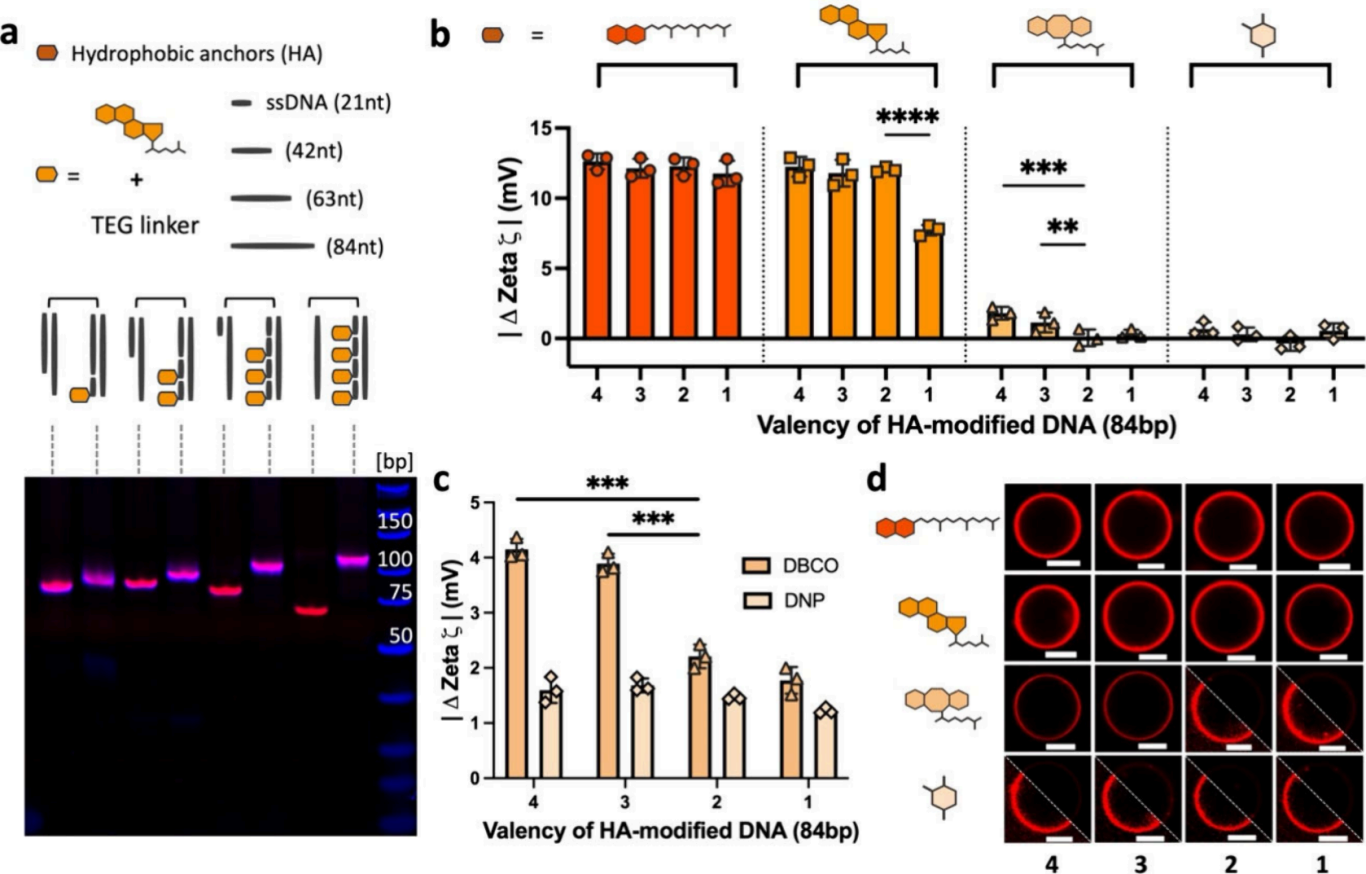
Multivalent hydrophobicity. (a) Schematic drawing illustrating the design of multivalent 84 bp DNA duplexes (e.g., cholesterol conjugates) and their PAGE gel analysis, categorized by monovalent, bivalent, trivalent, and tetravalent structures in the presence and absence of anchored strand (full analysis in Figure S5). (b) ζ potential measurement of the various multivalent-anchored DNA nanostructures incubated with DMPC LUVs at 37 °C (lipid phase). (c) Change in ζ potential of multivalent DBCO-modified and DNP-modified DNA nanostructure with doubled DNA molar ratio to lipids. Both error bars obtained from three independent measurements, each consisting of at least 15 subruns. Statistical significance was assessed using unpaired *t* test (**p < 0.01, ***p < 0.001, ****p < 0.0001). (d) Representative micrographs showing the effect of multivalency on the modified DNA incubated with POPC GUV (liquid phase) at 37 °C, where the laser settings were standardized based on the images having the highest intensity (i.e., 4-α-tocopherol dsDNA). It is noted that the attachment for one- and two-DBCO and DNP conjugates are shown with 3× brightness in the lower half of the triangle for visualization purposes. Scale bar: 10 μm.

To obtain a more detailed look at the valency effect for the weaker anchors, we increased the negative charges attached to the membrane by doubling the DNA/lipid molar ratio (Table S3). This doubled the absolute ζ change for three and four DBCO modifications, again confirming that three DBCO anchors provide sufficient anchoring strength to measure a robust signal. For DNP, no multivalent effects were observed (Figure 3c). Representative micrographs of all multivalent DNA conjugates were qualitatively compared in Figure 3d. Assuming that the 4-α-toco conjugate exhibits maximum binding tendency, we normalized all intensities to this sample, Figure S6a,b. The data highlight stronger attachment of 2-Chol and 4/3-DBCO modified duplexes compared to 1-Chol and 1/2-DBCO modified duplexes, respectively. Specifically, the results were categorized and normalized by each anchor to individually assess how multivalency affects their performance (Figure S6c), especially on multivalent Chol and DBCO conjugates, which is consistent with the results obtained from the zeta potential measurements.

Akin to the binding affinity between ligand–receptor couples, multivalency could enhance the overall hydrophobicity when anchors are multimerized. While a solitary α-tocopherol modification already reaches saturation in terms of DNA/membrane binding, increasing the number of DBCO anchors showed at least three are required for stable binding. This provides an additional method to modulate a more dynamic range of DNA–lipid interactions beyond merely using a series of strong anchors. This approach could prevent undesired aggregation of highly hydrophobic tags, such as cholesterol, which has been reported to compromise the structural integrity of modified DNA nanostructures,^17^ thus preserving the primary advantage offered by DNA nanotechnology.^29^

Besides controlled multimerization, DNA provides a unique opportunity to explore the effect of structural rigidity and spatial anchor orientation on overall binding yield. We examined both effects using the 4-DBCO system, as membrane interactions with this assembly were most affected by the valency of the anchor. The rigidity of DNA constructs affects the entropic penalty of binding, as increased flexibility indicates a higher loss of degrees of freedom upon binding.^30^ Two constructs were designed: a linear form (DBCO-L) and a tetrapod form (DBCO-TP), as illustrated in Figure 4a. To modulate the flexibility, single-stranded sections were introduced between the dsDNA domains with DBCO functionalization. This resulted in flexible linear (FL) and flexible tetrapod (FTP) constructs, respectively.

**Figure 4:**
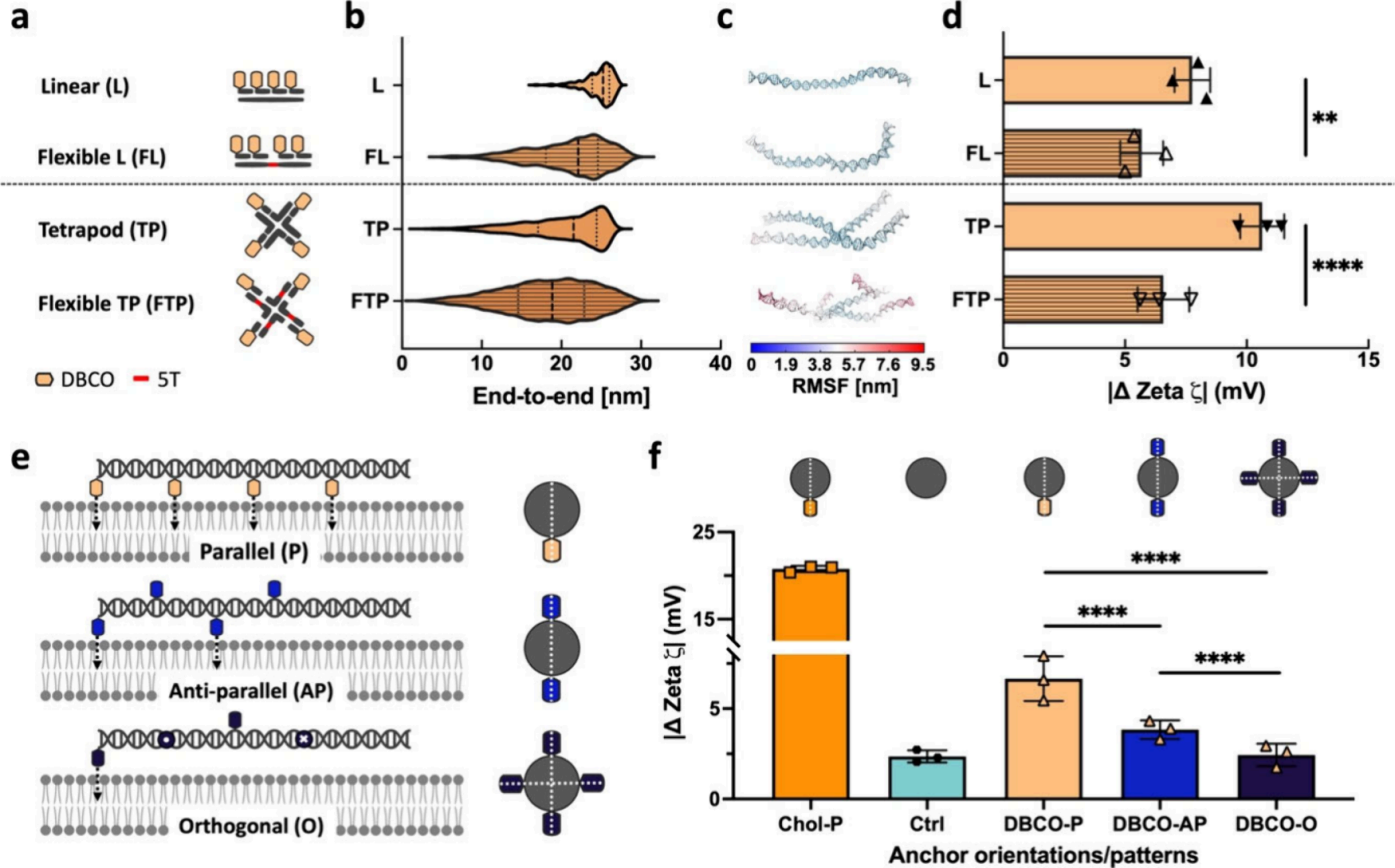
Flexibility and orientation contribute to tuning of interactions (a) Schematic overview of the library of rigid/flexible DNA constructs, in which the single-stranded regions are highlighted in red. (b) Violin plots of end-to-end distances for linear (L), flexible linear (FL), tetrapod (TP), and flexible tetrapod (FTP) structures acquired from coarse-grained simulations. (c) Corresponding last simulation frames, mapping root-mean-square-fluctuations (RMSF). (d) ζ potential analysis demonstrating the effect of structural flexibility. Error bar derived from three independent measurements. (e) Schematic presentation showing the conformational design of anchor orientation interacting with the lipid membrane, defining the number of effective DBCO anchors along with the cross-section view. (f) Changes in ζ potential measured by incubating DNA duplexes in a variety of configurations with the DMPC LUV in the liquid phase, where the error bar represents the standard deviation from three measurements, each consisting of at least 15 subruns. Statistical significance was assessed using an unpaired *t* test (**p < 0.01, ****p < 0.0001).

To quantitatively represent their conformational flexibility, we measured the end-to-end distances for each structure through coarse-grained oxDNA simulations.^31−34^ The widths of these distributions serve as indicators of their conformational flexibility (Figure 4b). Using the same model, we further examined their flexibility by mapping their root-mean-square fluctuations (RMSF), as depicted in Figure 4c. The wider distributions and greater RMSF values confirm that constructs with ssDNA domains exhibit an increased flexibility of the positioned anchors. Both flexible variants show a reduced interaction with the lipid membrane, as evidenced by a significantly smaller shift in ζ potential values (Figure 4d). Of note, the results between two nanostructures are only internally comparable, as the ζ potential value is determined not only by the number of bound nanostructures but also by the number of charges they withhold.

Finally, we decided to investigate the level of fine-tuning that we could achieve through the DNA backbone. The DNA double helix completes a single full turn approximately every 10.5 bp,^35^ allowing for precise positioning of the anchors along the reference axis. We designed DBCO-anchored DNA duplexes (84 bp) with the anchors positioned in parallel (P), antiparallel (AP), and orthogonal (O) configurations (Figure S8), as schematically depicted in Figure 4e. DBCO-P presents four anchors in the same side that can simultaneously interact with the membrane, indicating an effective valency of 4-DBCO that contributes to increased overall hydrophobicity. In absence of structural flexibility, DBCO-O and DBCO-AP configurations will reduce the effective valency to 1 and 2, respectively, due to the spatial constraints of anchors available to the membrane. Indeed, DBCO-P exhibited the strongest binding, with the interaction quickly becoming weaker for DBCO-AP and comparable to that of monomeric DBCO for the DBCO-O configuration (Figure 4f).

Based on the inherent rigidity of dsDNA and our previous assumption of a two-dimensional interface, we demonstrate how the hydrophobic-mediated DNA–lipid interaction can be further tuned through manipulating the spatial orientation of anchors. This emphasizes the correlation between structural considerations and effective valency on very short length scales, which should be considered in the experimental design of functional DNA-based biomaterials.

In this study, we aimed to elucidate the fundamental principles governing the modified DNA/lipid interactions, focusing on how these interactions are influenced by hydrophobicity, multivalency, and DNA structural design. While the interaction is predominantly driven by hydrophobicity of the modified DNA, the concept of multivalency can transform relatively weak anchors into stronger assemblies. Additionally, we illustrated that the structural design of DNA, including flexibility and rotation along the DNA axis, provides a broader spectrum of tunable parameters. These control knobs are only made available with the use of an anchor weaker than the standard go-to hydrophobic modification of cholesterol. Where the dynamic system is of higher priority than the strength of attachment, multivalency and a vast library of hydrophobic molecules should be in the DNA nanoengineer’s toolbox.

We demonstrated that effective attachment to lipid membranes in the liquid phase occurs only when anchors are sufficiently hydrophobic. For zwitterionic membranes in the gel phase, DNA–lipid interactions were previously shown to experience strong electrostatic bridging.^22^ Decoupling of electrostatic and hydrophobic effects in such systems is worth a future investigation. Our current study included a small series of readily available molecules. With the development of DNA modification, systematic studies comparing other hydrophobic moieties, for example poly(propylene oxide) (PPO)^36^ or porphyrin,^37^ and alkyne chains^38^ should be considered. This would build toward a framework for designing and predicting DNA biomaterial interactions with lipid membranes, highlighting their versatility and reliability as molecular tools for controlling modified DNA/lipid interactions.

In summary, our investigation into the hydrophobic-mediated DNA–lipid interaction has provided a comprehensive understanding of the various design strategies toward programmable DNA/lipid interfaces. The parameters discussed in this work expand the hydrophobicity toolbox, guiding the design of tailored DNA-based nanostructures with precise and dynamic control over their interactions with model membranes. In future studies, these parameters can be further evaluated in more complex systems, including biofilms and live cells. This precise understanding of interactions with biological membranes is key to optimizing the performance of DNA-based nanotherapeutics and devices.

## Supporting Information Available

Experimental details, materials, and methods (PDF)

## References

[ref1] Felgner P. L.; Gadek T. R.; Holm M.; Roman R.; Chan H. W.; Wenz M.; Northrop J. P.; Ringold G. M.; Danielsen M. Lipofection: A Highly Efficient, Lipid-Mediated DNA-Transfection Procedure. Proc. Natl. Acad. Sci. U. S. A. 1987, 84 (21), 7413–7417. 10.1073/pnas.84.21.7413. 2823261 PMC299306

[ref2] Abbott N. L.; Jewell C. M.; Hays M. E.; Kondo Y.; Lynn D. M. Ferrocene-Containing Cationic Lipids: Influence of Redox State on Cell Transfection. J. Am. Chem. Soc. 2005, 127 (33), 11576–11577. 10.1021/ja054038t. 16104714

[ref3] Ewert K. K.; Evans H. M.; Zidovska A.; Bouxsein N. F.; Ahmad A.; Safinya C. R. A Columnar Phase of Dendritic Lipid– Based Cationic Liposome– DNA Complexes for Gene Delivery: Hexagonally Ordered Cylindrical Micelles Embedded in a DNA Honeycomb Lattice. J. Am. Chem. Soc. 2006, 128 (12), 3998–4006. 10.1021/ja055907h. 16551108

[ref4] Buck J.; Grossen P.; Cullis P. R.; Huwyler J.; Witzigmann D. Lipid-Based DNA Therapeutics: Hallmarks of Non-Viral Gene Delivery. ACS Nano 2019, 13 (4), 3754–3782. 10.1021/acsnano.8b07858. 30908008

[ref5] Pardi N.; Hogan M. J.; Porter F. W.; Weissman D. mRNA Vaccines — a New Era in Vaccinology. Nat. Rev. Drug Discovery 2018, 17 (4), 261–279. 10.1038/nrd.2017.243. 29326426 PMC5906799

[ref6] Francis J. E.; Skakic I.; Smooker P. M. Design and Preparation of Solid Lipid Nanoparticle (SLN)-Mediated DNA Vaccines. Vaccine Des. Methods Protoc. Vol. 3 Resour. Vaccine Dev 2022, 2412, 355–366. 10.1007/978-1-0716-1892-9_18. 34918255

[ref7] Bolhassani A. Lipid-Based Delivery Systems in Development of Genetic and Subunit Vaccines. Mol. Biotechnol. 2023, 65 (5), 669–698. 10.1007/s12033-022-00624-8. 36462102 PMC9734811

[ref8] Dimova R.; Marques C. The Giant Vesicle Book; CRC Press, 2019.

[ref9] Forni G.; Mantovani A. COVID-19 Vaccines: Where We Stand and Challenges Ahead. Cell Death Differ. 2021, 28 (2), 626–639. 10.1038/s41418-020-00720-9. 33479399 PMC7818063

[ref10] Langecker M.; Arnaut V.; List J.; Simmel F. C. DNA Nanostructures Interacting with Lipid Bilayer Membranes. Acc. Chem. Res. 2014, 47 (6), 1807–1815. 10.1021/ar500051r. 24828105

[ref11] Shen Q.; Grome M. W.; Yang Y.; Lin C. Engineering Lipid Membranes with Programmable DNA Nanostructures. Adv. Biosyst. 2020, 4 (1), 1900215 10.1002/adbi.201900215. 31934608 PMC6957268

[ref12] Langecker M.; Arnaut V.; Martin T. G.; List J.; Renner S.; Mayer M.; Dietz H.; Simmel F. C. Synthetic Lipid Membrane Channels Formed by Designed DNA Nanostructures. Science 2012, 338 (6109), 932–936. 10.1126/science.1225624. 23161995 PMC3716461

[ref13] Thomsen R. P.; Malle M. G.; Okholm A. H.; Krishnan S.; Bohr S. S.-R.; Sørensen R. S.øl.; Ries O.; Vogel S.; Simmel F. C.; Hatzakis N. S.; Kjems Jør. A Large Size-Selective DNA Nanopore with Sensing Applications. Nat. Commun. 2019, 10 (1), 5655 10.1038/s41467-019-13284-1. 31827087 PMC6906287

[ref14] Akbari E.; Mollica M. Y.; Lucas C. R.; Bushman S. M.; Patton R. A.; Shahhosseini M.; Song J. W.; Castro C. E. Cell-Membrane Engineering: Engineering Cell Surface Function with DNA Origami (Adv. Mater. 46/2017). Adv. Mater. 2017, 29 ( (46), ). 10.1002/adma.201770328. PMC5739518 29027713

[ref15] Li J.; Xun K.; Pei K.; Liu X.; Peng X.; Du Y.; Qiu L.; Tan W. Cell-Membrane-Anchored DNA Nanoplatform for Programming Cellular Interactions. J. Am. Chem. Soc. 2019, 141 (45), 18013–18020. 10.1021/jacs.9b04725. 31626550

[ref16] Banchelli M.; Betti F.; Berti D.; Caminati G.; Bombelli F. B.; Brown T.; Wilhelmsson L. M.; Nordén B.; Baglioni P. Phospholipid Membranes Decorated by Cholesterol-Based Oligonucleotides as Soft Hybrid Nanostructures. J. Phys. Chem. B 2008, 112 (35), 10942–10952. 10.1021/jp802415t. 18693696

[ref17] Ohmann A.; Göpfrich K.; Joshi H.; Thompson R. F.; Sobota D.; Ranson N. A.; Aksimentiev A.; Keyser U. F. Controlling Aggregation of Cholesterol-Modified DNA Nanostructures. Nucleic Acids Res. 2019, 47 (21), 11441–11451. 10.1093/nar/gkz914. 31642494 PMC6868430

[ref18] Morzy D.; Rubio-Sánchez R.; Joshi H.; Aksimentiev A.; Di Michele L.; Keyser U. F. Cations Regulate Membrane Attachment and Functionality of DNA Nanostructures. J. Am. Chem. Soc. 2021, 143 (19), 7358–7367. 10.1021/jacs.1c00166. 33961742 PMC8154537

[ref19] Banchelli M.; Gambinossi F.; Durand A.; Caminati G.; Brown T.; Berti D.; Baglioni P. Modulation of Density and Orientation of Amphiphilic DNA on Phospholipid Membranes. II. Vesicles. J. Phys. Chem. B 2010, 114 (21), 7348–7358. 10.1021/jp100731c. 20446699

[ref20] Krishnan S.; Ziegler D.; Arnaut V.; Martin T. G.; Kapsner K.; Henneberg K.; Bausch A. R.; Dietz H.; Simmel F. C. Molecular Transport through Large-Diameter DNA Nanopores. Nat. Commun. 2016, 7 (1), 12787 10.1038/ncomms12787. 27658960 PMC5036142

[ref21] Jones S. F.; Joshi H.; Terry S. J.; Burns J. R.; Aksimentiev A.; Eggert U. S.; Howorka S. Hydrophobic Interactions between DNA Duplexes and Synthetic and Biological Membranes. J. Am. Chem. Soc. 2021, 143 (22), 8305–8313. 10.1021/jacs.0c13235. 34015219 PMC8193631

[ref22] Morzy D.; Tekin C.; Caroprese V.; Rubio-Sánchez R.; Di Michele L.; Bastings M. M. C. Interplay of the Mechanical and Structural Properties of DNA Nanostructures Determines Their Electrostatic Interactions with Lipid Membranes. Nanoscale 2023, 15 (6), 2849–2859. 10.1039/D2NR05368C. 36688792 PMC9909679

[ref23] Kurz A.; Bunge A.; Windeck A.-K.; Rost M.; Flasche W.; Arbuzova A.; Strohbach D.; Muller S.; Liebscher J.; Huster D.; Herrmann A. Lipid-anchored Oligonucleotides for Stable Double-helix Formation in Distinct Membrane Domains. Angew. Chem., Int. Ed. 2006, 45 (27), 4440–4444. 10.1002/anie.200600822. 16789049

[ref24] Rubio-Sánchez R.; Barker S. E.; Walczak M.; Cicuta P.; Michele L. D. A Modular, Dynamic, DNA-Based Platform for Regulating Cargo Distribution and Transport between Lipid Domains. Nano Lett. 2021, 21 (7), 2800–2808. 10.1021/acs.nanolett.0c04867. 33733783 PMC8050828

[ref25] Chemicalize - Instant Cheminformatics Solutions. Chemicalize. https://chemicalize.com (accessed March 14, 2024).

[ref26] Smith M. C.; Crist R. M.; Clogston J. D.; McNeil S. E. Zeta Potential: A Case Study of Cationic, Anionic, and Neutral Liposomes. Anal. Bioanal. Chem. 2017, 409, 5779–5787. 10.1007/s00216-017-0527-z. 28762066

[ref27] Fasting C.; Schalley C. A.; Weber M.; Seitz O.; Hecht S.; Koksch B.; Dernedde J.; Graf C.; Knapp E.; Haag R. Multivalency as a Chemical Organization and Action Principle. Angew. Chem., Int. Ed. 2012, 51 (42), 10472–10498. 10.1002/anie.201201114. 22952048

[ref28] Bila H.; Paloja K.; Caroprese V.; Kononenko A.; Bastings M. M. Multivalent Pattern Recognition through Control of Nano-Spacing in Low-Valency Super-Selective Materials. J. Am. Chem. Soc. 2022, 144 (47), 21576–21586. 10.1021/jacs.2c08529. 36383954 PMC9716526

[ref29] Comberlato A.; Koga M. M.; Nüssing S.; Parish I. A.; Bastings M. M. Spatially Controlled Activation of Toll-like Receptor 9 with DNA-Based Nanomaterials. Nano Lett. 2022, 22 (6), 2506–2513. 10.1021/acs.nanolett.2c00275. 35266392 PMC8949768

[ref30] Martinez-Veracoechea F. J.; Leunissen M. E. The Entropic Impact of Tethering, Multivalency and Dynamic Recruitment in Systems with Specific Binding Groups. Soft Matter 2013, 9 (12), 3213–3219. 10.1039/c3sm27766f.

[ref31] Poppleton E.; Romero R.; Mallya A.; Rovigatti L.; Šulc P. OxDNA.Org: A Public Webserver for Coarse-Grained Simulations of DNA and RNA Nanostructures. Nucleic Acids Res. 2021, 49 (W1), W491–W498. 10.1093/nar/gkab324. 34009383 PMC8265093

[ref32] Rovigatti L.; Šulc P.; Reguly I. Z.; Romano F. A. Comparison between Parallelization Approaches in Molecular Dynamics Simulations on GPUs. J. Comput. Chem. 2015, 36 (1), 1–8. 10.1002/jcc.23763. 25355527

[ref33] Snodin B. E. K.; Randisi F.; Mosayebi M.; Šulc P.; Schreck J. S.; Romano F.; Ouldridge T. E.; Tsukanov R.; Nir E.; Louis A. A.; Doye J. P. K. Introducing Improved Structural Properties and Salt Dependence into a Coarse-Grained Model of DNA. J. Chem. Phys. 2015, 142 (23), 234901 10.1063/1.4921957. 26093573

[ref34] Humphrey W.; Dalke A.; Schulten K. VMD: Visual Molecular Dynamics. J. Mol. Graph. 1996, 14 (1), 33–38. 10.1016/0263-7855(96)00018-5. 8744570

[ref35] Travers A.; Muskhelishvili G. DNA Structure and Function. FEBS J. 2015, 282 (12), 2279–2295. 10.1111/febs.13307. 25903461

[ref36] Rodríguez-Franco H. J.; Weiden J.; Bastings M. M. Stabilizing Polymer Coatings Alter the Protein Corona of DNA Origami and Can Be Engineered to Bias the Cellular Uptake. ACS Polym. Au 2023, 3 (4), 344–353. 10.1021/acspolymersau.3c00009. 37576710 PMC10416322

[ref37] Börjesson K.; Lundberg E. P.; Woller J. G.; Nordén B.; Albinsson B. Soft-Surface DNA Nanotechnology: DNA Constructs Anchored and Aligned to Lipid Membrane. Angew. Chem., Int. Ed. Engl. 2011, 50 (36), 8312 10.1002/anie.201103338. 21761537 PMC3193381

[ref38] Gut I. G.; Beck S. A Procedure for Selective DNA Alkylation and Detection by Mass Spectrometry. Nucleic Acids Res. 1995, 23 (8), 1367–1373. 10.1093/nar/23.8.1367. 7753628 PMC306863

