# Supplementary material for: Modulating the DNA/Lipid Interface through Multivalent Hydrophobicity
Source: Nano Lett. 2024 Jul 26;24(36):11210–6. doi: 10.1021/acs.nanolett.4c02564 (PMC11403765; doi:10.1021/acs.nanolett.4c02564)
Supplement: Supplementary file 1 — nl4c02564_si_001.pdf [file nl4c02564_si_001.pdf]

Supporting information

Modulating the DNA/lipid interface through multivalent hydrophobicity

Siu Ho Wong[a], Sarina Nicole Kopf[a], Vincenzo Caroprese[a], Yann Zosso[a], Diana Morzy[a], and Maatje M.C Bastings*[a,b]

[a]Programmable Biomaterials Laboratory, Institute of Materials, School of Engineering, Ecole Polytechnique Fédérale Lausanne, Lausanne, 1015, Switzerland

[b]bInterfaculty Bioengineering Institute, School of Engineering, Ecole Polytechnique Fédérale Lausanne, Lausanne, 1015, Switzerland

E-mail: maartje.bastings@epfl.ch

Methods

All chemical reagents were acquired from Sigma Aldrich, unless otherwise specified. All hydrophobic tag-TEG-DNA oligonucleotides were customized and purchased from Eurogentec with high-performance liquid chromatography (HPLC) and electrospray ionization (ESI) mass spectrometry for quality control, while all other oligonucleotides were provided by Integrated DNA Technologies with standard PAGE purification, Inc. Lipids were obtained from Avanti® Polar Lipids. All chemical structures were illustrated in Chemdraw. Graphs illustrating the results and statistical analyses were produced and performed using GraphPad Prism version 10.0.0 for Mac.

<u>**DNA Design and Nanostructure Assembly:**</u> To avoid the formation of secondary structures and to ensure sufficient yield of folding, all DNA nanostructures were designed and analyzed with the NUPACK suite[1,2]. All strands were dissolved in ultrapure Ultrapure™ DNase/RNase-Free distilled water to a final concentration of 100 µM. While unmodified strands were stored at 4°C, the modified ones stored at -20 °C.

To assemble the designed structures, corresponding strands were mixed to a final concentration of 1 µM in TE buffer (10 mM Tris, 1 mM EDTA, pH 8.0). Notably, hydrophobically modified strands were vortexed vigorously at 4°C to minimize the intermolecular hydrophobic

interactions. Additionally, all DNA duplexes were prepared in the buffer containing 1 mM magnesium concentration. Mixture was finally annealed in a thermocycler programmed at 80°C for 5 min, and subsequently cooled down from 60°C to 20°C with the rate of 1°C/min. The resulting folded structures were stored at 4°C. DNA sequences and details of modifications are summarized in **Table S1**.

Large and Giant Lipids Vesicle: Large unilamellar vesicles (LUVs) employed for the zeta (ζ) potential measurements were prepared using a commercially available extruder with the extrusion protocol provided by Avanti® Polar Lipids. Whatman® Nucleopore Track-Etched Membranes (100 nm) and Whatman® Filter Supports were applied to define the LUVs sizes. The employed DMPC (1,2-dimyristoyl-sn-glycero-3-phosphocholine) lipids layer was formed in a round-bottom fluask, and was rehydrated in 200 mM sucrose mixed with TE buffer (pH = 7.5), followed by the extrusion performing at 40 °C to guarantee bilayer fluidity. The corresponding size distribution of LUVs was measured with dynamic light scattering (DLS) measurement for quality control (**Figure S2**). All LUVs were stored at 4 °C and consumed only within a week.

Giant unilamellar vesicles (GUVs) used in the imaging were prepared with electroformation, following the established protocol[3–5]. The employed lipids were DPPC (1,2-dipalmitoyl-sn-glycero-3-phosphocholine) used in **Figure. 1** and POPC (1-palmitoyl-2-oleoyl-glycero-3-phosphocholine) with an addition of fluorescent-labelled NBD-PC lipids (1-palmitoyl-2-{6-[(7-nitro-2-1,3-benzoxadiazol-4-yl)amino]hexanoyl}-sn-glycero-3-phosphocholine) in a molar ratio of 200:1. In short, indium tin oxide (ITO) slides were cleaned through 15-minute sonication cycles with isopropanol/MiliQ water, followed by drying under a gentle nitrogen flow. Subsequently, the slides were heated to 50 °C, and 80 µL of lipids of interest (25 mg/mL) were evenly spread on the conducting side using a clean glass coverslip. After resting for 1 hour under vacuum in desiccator, they were assembled into an electroformation chamber by coupling them with an approximately 2 mm thick polydimethylsiloxane (PDMS) spacer containing 800 µL of 200 mM sucrose mixed with TE buffer (pH = 7.5) was used as a buffer with the osmolality of around 200 mOsm. Theses chambers were connected to a frequency generator with clamps, and exposed to a sinusoidal alternating current (AC) with a voltage amplitude of 2V and a frequency of 10 Hz for the first 2 hours and then 2 Hz for the next hour.

In the case of electroformation for DPPC (1,2-dihexadecanoyl-sn-glycero-3-phosphocholine) GUVs with a high-transition temperature, the process was performed at 60 °C to ensure bilayer fluidity. The transition temperature (i.e., chain melting temperature) of all employed lipids are shown in **Table S2**. Obtained GUVs were stored at 4 °C and used within a week.

Phosphatidylcholine Assay: The concentration of synthesized LUVs/GUVs was determined using a commercially available phosphatidylcholine (PC) assay kit *via* fluorometric detection. Following the manufacturer's technical bulletin, fluorometric products (λ_{ex} = 535/λ_{em} = 587 nm) was acquired for the PC standard and samples, diluted in a reaction mixture containing assay buffer, hydrolysis enzyme, fluorescent peroxidase substrate, and development mix. A standard curve was plotted with the provided PC standard solution. It is noted that a new standard curve must be set up along with each concentration measurement. The calculation was finally carried out by subtracting the background values from all readings to estimate the concentration of PC in the synthesized vesicles with the equation: $S_a/S_v = C$, where S_a is the amount of PC in unknown sample (nmole) from standard curve, S_v is the sample volume (µL) added into the wells, and C is the calculated concentration of PC in the sample. The sample concentrations were standardized to ensure the same lipid/DNA molar ratio across each replicate.

Confocal Microscopy Imaging: Glass slides, cleaned with 15 min sonication cycles (absolute ethanol/isopropanol/MiliQ water) were pretreated with bovine serum albumin (BSA) at 0.1% (w/v) and assembled with the Grace Bio-Labs FlexWell™ (6.5×6.5×.3.2 mm) to prepare the sample chamber. For imaging, the confocal images for GUVs were captured on a Leica SP8 inverted microscope using a HC PL APO 20× /0.75 dry objective at 1.5x zoom factor, line average - 4, and 37 °C (unless otherwise specified) within a stage top chamber (Okolab). The standardized GUVs and desired DNA concentration were mixed in 50 µL of 200 mM glucose with TE buffer (pH = 7.5) in 1 mM Mg^{2+}. After incubating for 5 min, NBD (excitation - 467 nm; emission range - 500–570 nm) and Cy5 (excitation - 651 nm; emission range - 650–720 nm) were tracked using 488 nm and 638 nm solid state lasers for excitation. The laser and detector gain settings are individualized to each set of experiment, depending on the length and modification of DNA duplex to avoid saturation. The setting used in the qualitative comparison for non-modified DNA 63 bp duplex presented in **Figure 1b**, required a relatively

higher laser power and gain to observe the electrostatic DNA binding and dispersion for GUVs in both gel and liquid phase. All qualitative images presented were processed using FIJI with applied look-up table, display, and scale bar.

Image Acquisition, Processing and Analysis: Confocal micrographs of DNA-functionalized GUVs were used to assess the DNA binding based on the Cy5-labeled DNA intensity on the membranes. The screening and acquisition process followed the guiding criteria, where GUVs were imaged when: 1) at least 5 vesicles were big enough (>10 µm) in the field of view; 2) the vesicle of interest had similar sizes within the field of view; 3) no birght lipid aggregates were present in/within the membrane due to imperfect electroformation. While the criterion 1) suggests that Brownian motion was negligible within the acquistion time (of under a minute per image), the criterion 2) ensures their positions, so that they were mostly present in the conjugated focal plane. It is noted that the fluoresence siginal intensity of DNA were generally lower for vesicles that were out-of-focus. As for the Criterion 3), it was obversed that labeled DNA generally accumulated in the aggregated lipids (clumps), possibly due to reduced entropic costs from the immoblie lipid membrane. These should be taken in consideration for futher image processing and biased estimation of DNA nanostructure fluorescence. In all confocal experiments, at least 10 images were captured for each of three replicates corresponding to their experimental conditions.

The image processing is based on the user-assisted and python-based segmentation of the NBD channel for the lipid membrane. Briefly speaking, the custom-built python code loads each set of micrographs for both Cy5 DNA and NBD Lipid channels and applies a Gaussian (blurring) filter to facilitate segmentation and remove noise. Followed by binarization, multiple population otsu thresholding was performed with a closing operator. Importantly, vesicles, which were touching the border of the image and in close contact of one another, were excluded from the analysis. Subsequently, were processed with watershed skeletonization to skeletonize the final masks for segmentation.

For the image analysis, the positions and numbers obtained from the masks were stored in the data frame and applied to the NBD Cy5 channels to record the label, area, perimeter, circularity, centroid of the segmented vesciles as well as mean intensity and median intensity

of the DNA nanostructure. It is noted that the intensitiy proceeded with the subtraction of the background signal. After applying a circularity and out-of-focus filters as well as manual screening, the mean Cy5 (DNA) intensties of vesicle were normalized to the average intensity of the specific condition with the highest average intensity and plotted accordingly.

Dynamic Light Scattering and Zeta Potential: The measurements for LUVs were performed on a Zetasizer Nano ZS (Malvern Panalytical) at an excitation wavelength of 633 nm and a scattering angle fixed at 173°. Using a DTS1070 cell cuvette (Malvern Panalytical), each LUVs sample was first measured with size measurement for assessing its hydrodynamic radius in intensity profile (**Figure. S2**) as a quality control. The ζ potential measurement was performed with the desired DNA concentration and diluted LUVs in 1 mL of 200 mM sucrose with 1 mM Mg^{2+} mixed with TE buffer (pH = 7.5). Different molar ratios of DNA to LUV were described in **Table S3**, depending on the saturation curve measured for each set of experiments (**Figure. S9**). In **Figure 1**, the DMPC mixtures were separately incubated and measured at the temperature ranging from 15–35 °C after equilibrated for 5 min. The absolute changes in ζ potential (($|\Delta\zeta| = |\zeta_t - \zeta_0|$)) for all conjugates were obtained by subtracting the positive baseline of naked LUVs in the presence of 1 mM Mg^{2+}. All other measurements were performed at 37 °C with 5 min equilibration time of measuring temperature. Each replicate contained at least 3 measurements for each condition, each of at least 15 sub-runs. A table with the measured average zeta potential values is presented in **Table S4**.

Coarse-Grained Simulation: All initial configurations and topology files were prepared using the software cadnano[6] and converted to suitable formats through SNUPI[7,8]. For all structures examined, molecular dynamics (MD) simulations were performed using the oxDNA server[9], which leverages the CUDA-enhanced version of the simulation engine[10]. We employed the sequence-dependent oxDNA2[11] model for this study. Before initiating the production runs, a relaxation procedure recommended by the oxDNA documentation was applied. This involved conducting a Monte Carlo simulation for 10^5 steps, followed by an MD simulation for 10^7 steps, with all other parameters set according to the standard recommendations.

The production runs were executed at a temperature of 37°C and a salt concentration of 0.5 M, within an NVT ensemble. A time step of 10^{-3} was utilized, and configuration data were

captured every 5×10^6 steps. At least 954 configurations were sampled for each construct. Unless otherwise noted, all additional parameters adhered to the default settings provided by the oxDNA platform[9].

Post-simulation, the topologies and the associated trajectories containing the sampled configurations were respectively converted into PSF and MDCRD format using custom Python scripts. These files were then used for analysis using Virtual Molecular Dynamics (VMD)[12], supported by custom TCL scripts to streamline the process. The distances reported were determined based on the centers of mass of the base pairs of interest, after removing periodic boundary conditions.

Polyacrylamide Gel electrophoresis: Native PAGE was applied to confirm the expected folding of DNA structure designs. Gels were prepared with polyacrylamide at a concentration of 5 or 10%, 0.5× TBE (Tris-Borate-EDTA, pH 8.3) buffer. The mixture was casted to a thickness of 1 mm and allowed to set for 30 min. Subsequently, the gels were placed in an electrophoresis chamber and covered with 0.5× TBE. The DNA sample loading (30-150 ng), DNA reference ladders, and applied potential/time were individualized depending on the length of the DNA structures analyzed. The gel was finally imaged using a Bio-Rad ChemiDoc Imaging System for illumination.

Supplementary Figures

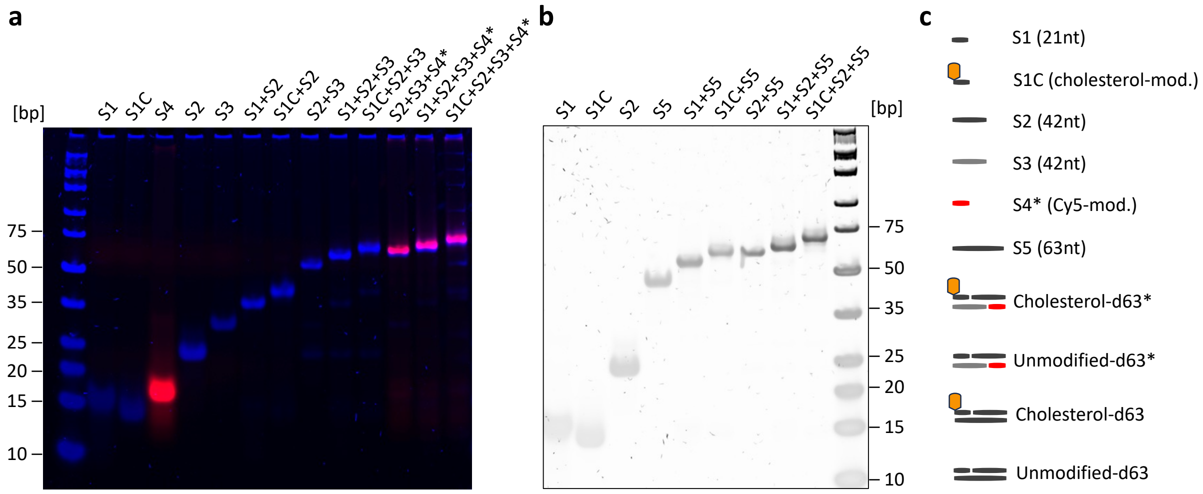

Figure S1. Polyacrylamide gel electrophoresis anaylsiss for stands used Figure 1. (a) Polyacrylamide gel electrophoresis analysis of unmodified and cholesterol modified 63-bp long DNA duplex labelled with Cy5 and (b) without Cy5 labels used in confocal experiment and zeta potential measurement, respectively. Signals were obtained in Cy5 and SYBR gold channels. (c) Schematic drawings illustrating the corresponding strands described in Table S1 and the assembled structures.

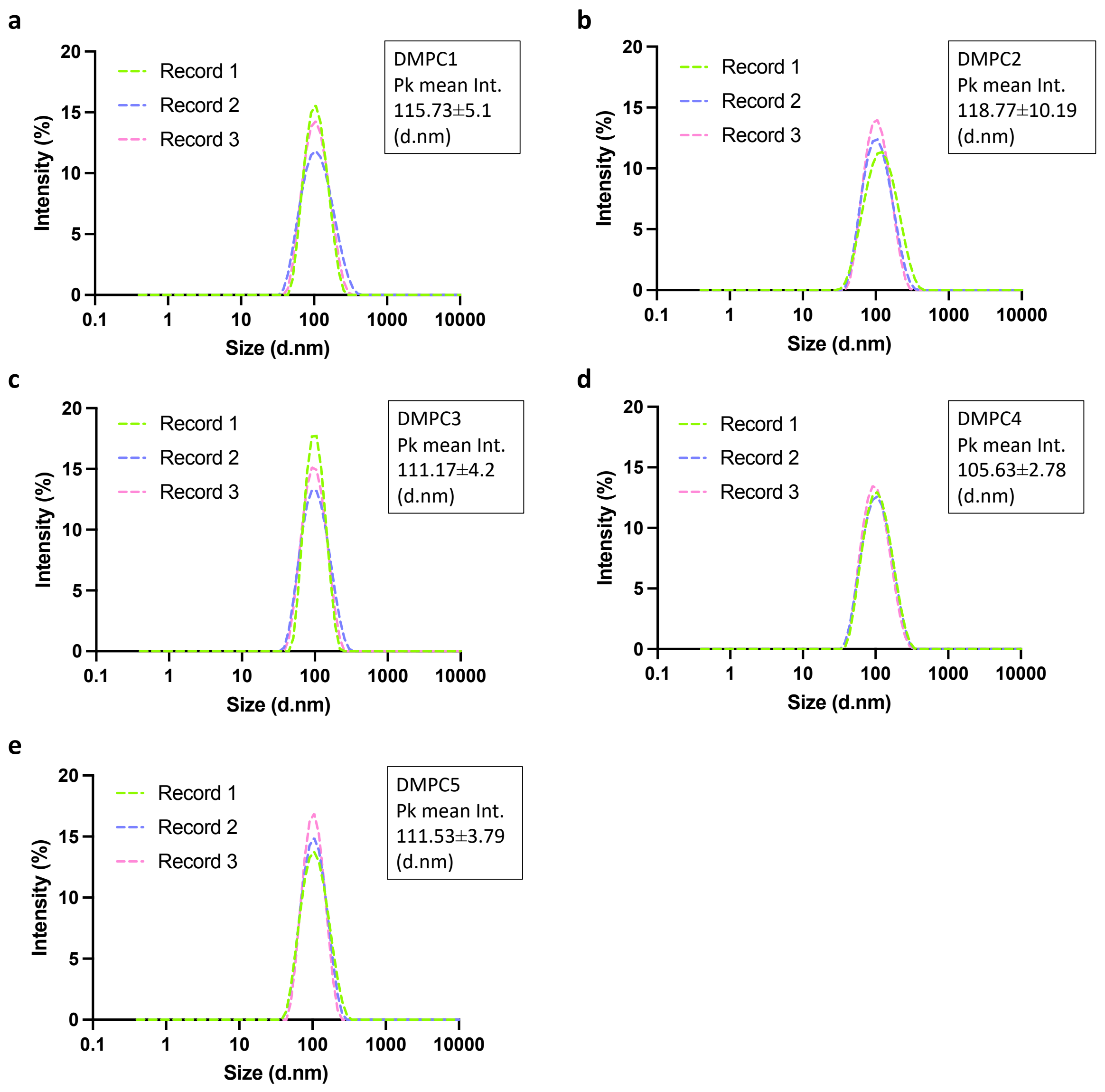

Figure S2. Quality control for LUVs. Size measurement obtained from dynamic light scattering of all extruded DMPC LUVs used in (a) Figure 1, (b) Figure 2, (c) Figure 3, and (d),(e), Figure 4, showing the average size and standard deviation from three independent replicates prior to zeta potential measurements. It is noted that all DMPC were freshly prepared prior to each replicate and proceed to ζ potential measurement.

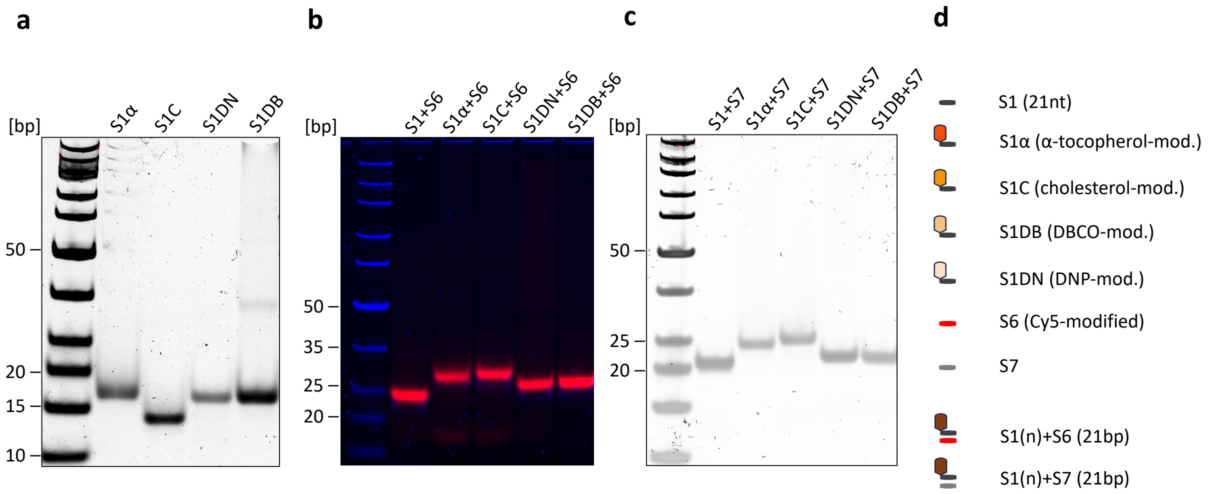

Figure S3. Polyacrylamide gel electrophoresis anaylsiss for stands used Figure 2. Polyacrylamide gel electrophoresis analysis of (a) hydrophobic anchored modified ssDNA as the anchor strands, as well as (b) 21-bp long dsDNA with Cy5 labels and (c) without Cy5 labels used in confocal experiment and zeta potential measurement, respectively. (d) Schematic drawings illustrating the corresponding strands described in Table S1 and the assembled structures.

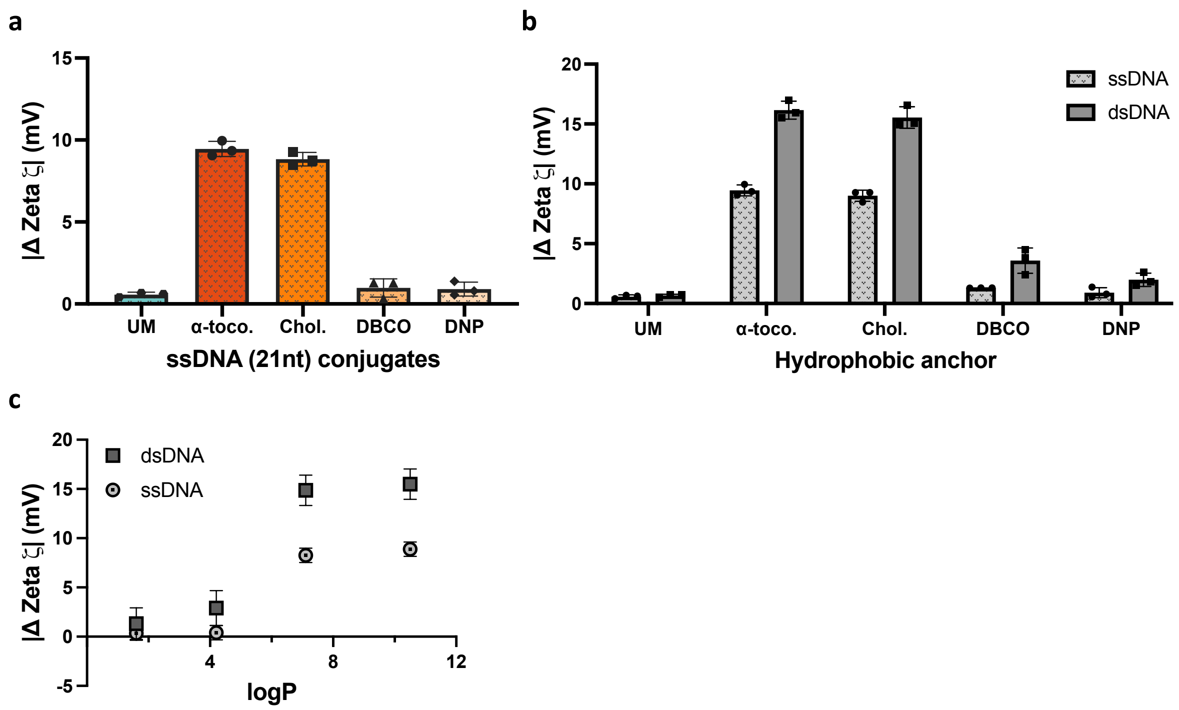

Figure S4. Additional experiemental results for Figure 2. (a) ζ potential measurement of anchored ssDNA attachment to DMPC LUV. (b) Comparison between the changes in ζ potential for hydrophobically modfiied ssDNA (21nt) and dsDNA (21bp). (c) The corresponding ζ potential values plotted against the hydrophobic scales presented by logP values. Error bar represents standard deviation from three measurements, each consisting of at least 15 sub-runs.

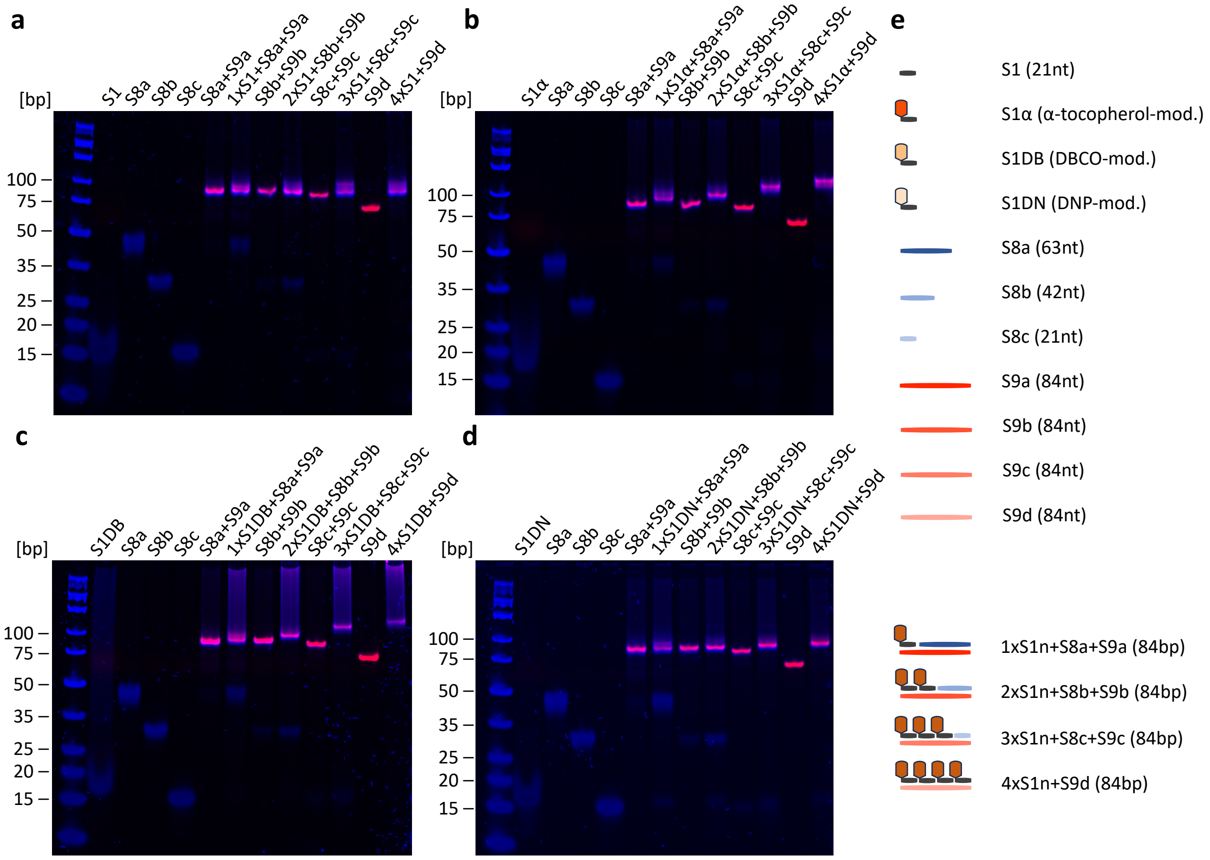

Figure S5. Polyacrylamide gel electrophoresis anaylsiss for stands used Figure 3. Polyacrylamide gel electrophoresis analysis of Cy5 labelled 84-bp long dsDNA with (a) unmodification, (b) α-tocopherol, (c) DBCO, and (d) DNP modfiications. (e) Schematic drawings illustrating the corresponding strands described in Table S1 and the assembled structures.

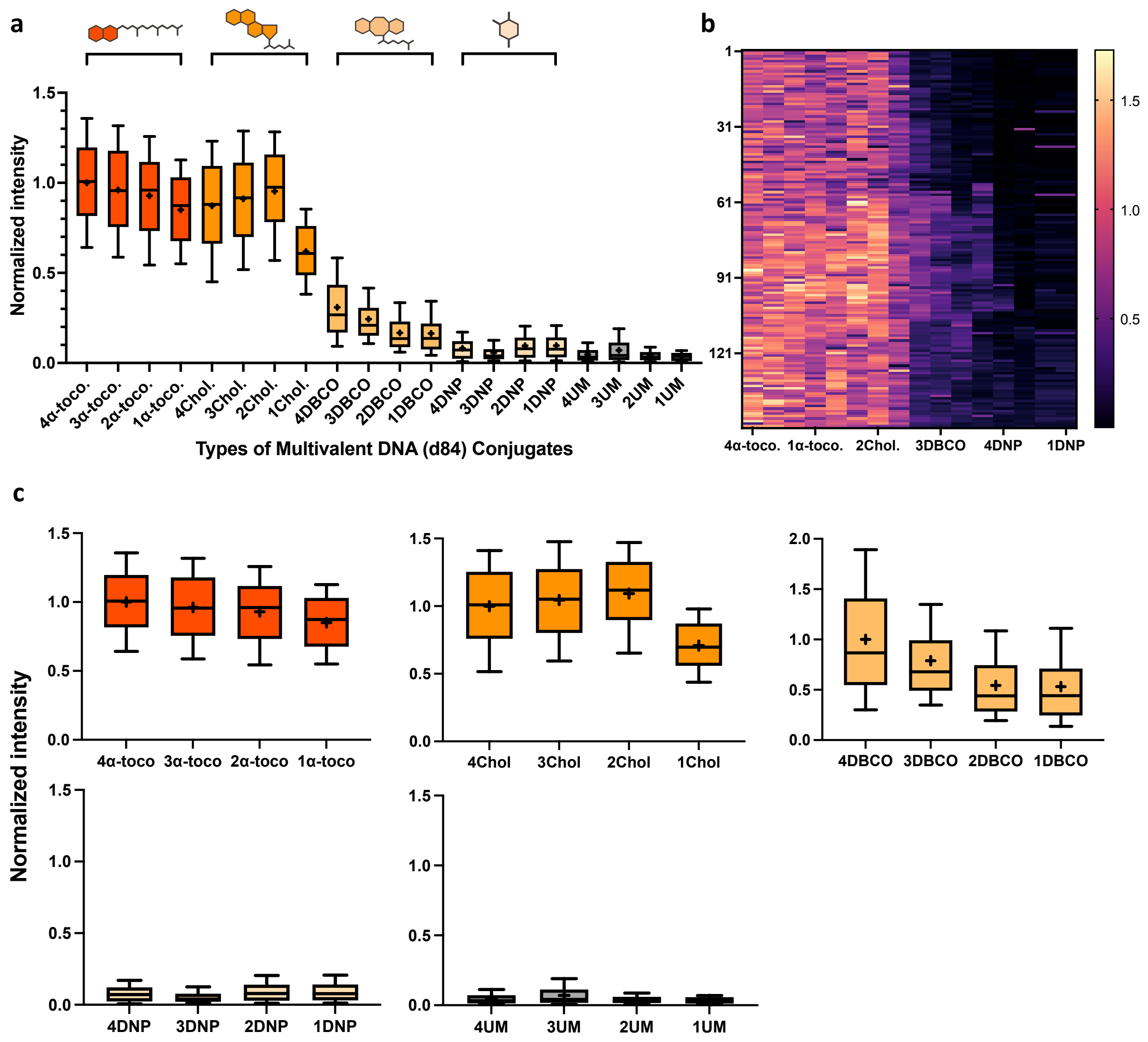

Figure S6. (a) Additional quantitative data retrieved from confocal images presented in Figure 3d. Confocal image data quantified by image processing, showing the effect of varying the multivalency from 4 to 1 on all hydrophobic anchors for comparison. The intensity for all conjugates is normalized based on the strongest interaction (i.e., four α-tocopherol modifications). (b) Fluorescent signals computed around POPC vesicles from 150 randomly selected data points from the processed images and plotted as normalized intensity. (c) Quantified data obtained from confocal images to show the effect of multivalency on indivdual hydrophobic anchor. It is noted that data are normalized within each group fro α-tocopherol, Cholesterol, and DBCO. Error bar represent whiskers.

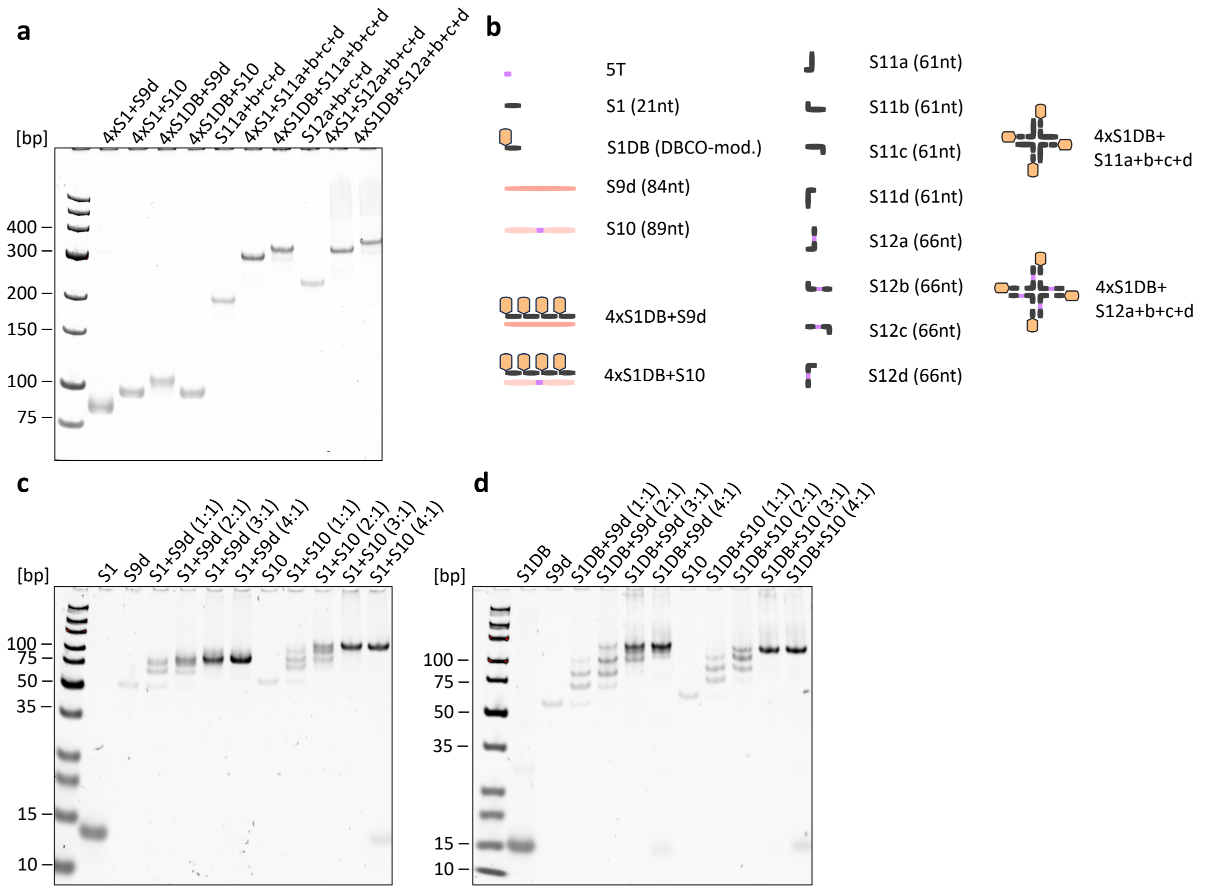

Figure S7. Polyacrylamide gel electrophoresis anaylsiss for stands used Figure 4a–d. Polyacrylamide gel electrophoresis analysis of (a) unmodified and DBCO modified Cy5 labelled 84-bp linear and flexible linear dsDNA complexes, as well as the assembled DNA terapods with four ummodified or DBCO modified anchor strands. (b) Schematic drawings illustrating the corresponding strands described in Table S1 and the assembled structures. In-depth analysis showing a complete assembly of (c) unmodified and (d) DBCO modified 84-bp dsDNA complexes with the adopted ratio of ummodified or DBCO anchored strands (S1 & S1DB) and based strands (S9d & S10).

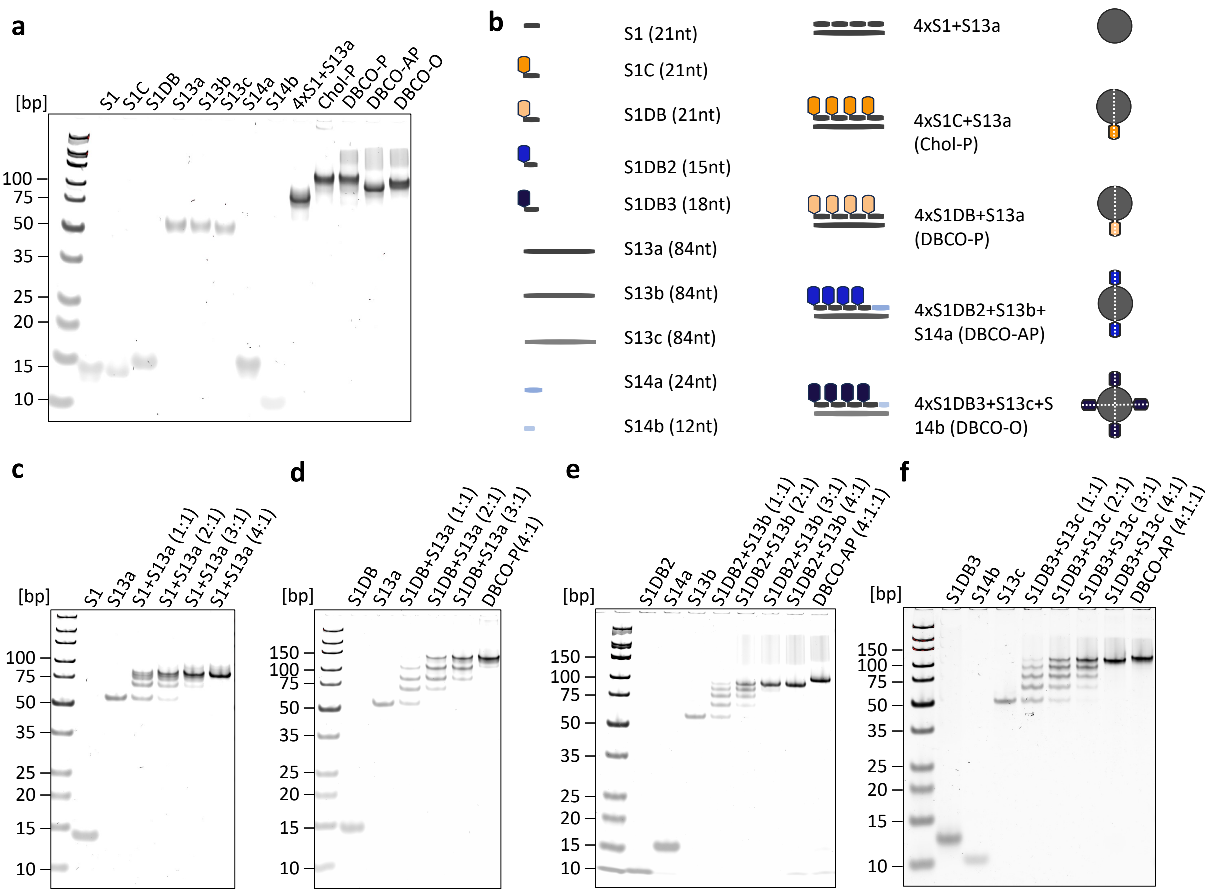

Figure S8. Polyacrylamide gel electrophoresis anaylsiss for stands used Figure 4e–f Polyacrylamide gel electrophoresis analysis of the DNA constructs and their components in different configurations, including parrallel (P), anti-parrallel (AP), and orthogonal (O). (b) Schematic drawings illustrating the corresponding strands described in Table S1 and the assembled structures. In-depth analysis showing a complete assembly of (c) unmodified 84-bp dsDNA, 84-bp dsDNA complexes with four DBCO anchors modification in (d) parrallel, (e) anti-parrallel, and (f) orthogonal in various molar ratios of anchor strands and base strands.

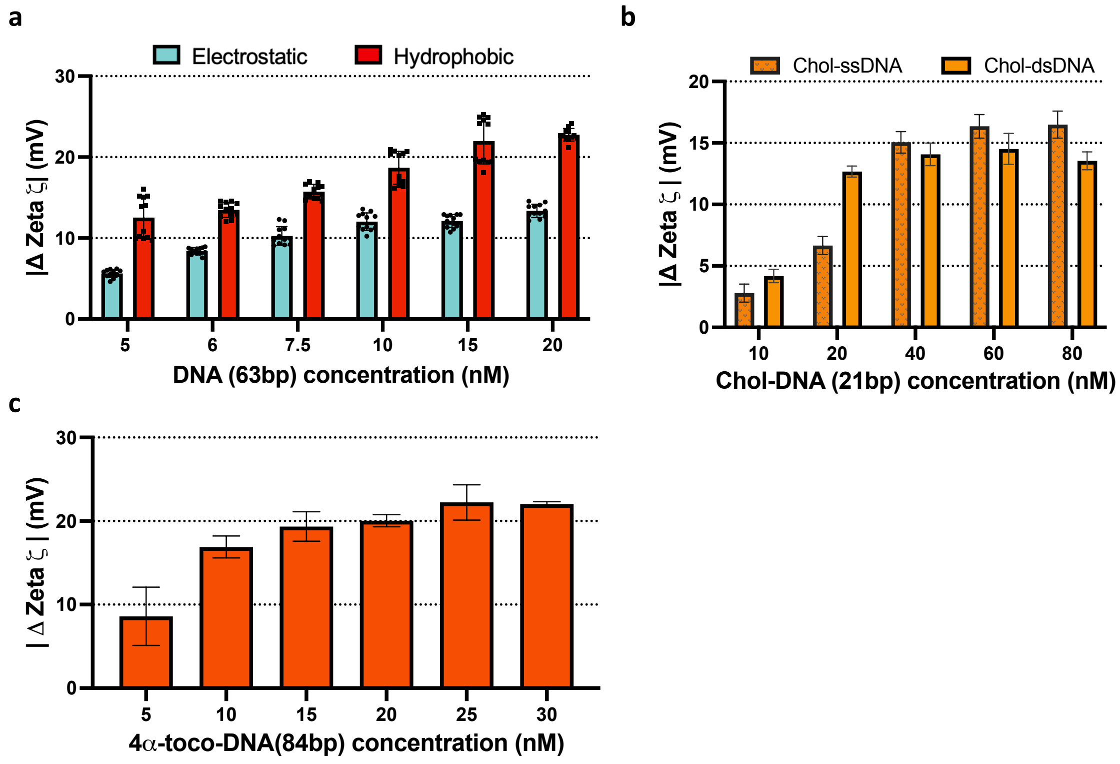

Figure S9. Additional experiemental data to determine the DNA concentration. Saturation curves primarily showing the concentration screening of (a) umnodified and cholesterol-modfiied 63 bp long DNA, (b) Cholesterol-modfied ssDNA (21nt) and dsDNA (21bp), and (c) 4α-tocopherol-modified 84 bp DNA, so as to determined the and molar ratio descibed in Table S3.

Supplementary Table

Table S1: DNA constructs and sequenecs. Sequences of oligonucleotides used to build the library of DNA duplexes presented in Figure 1- 4. L and Mod stands for length and modification, respectively.

Strand	Sequence	L.	Mod.	Used in
S1	GATGACGGTATAGCAAGTGTG	21	-	Fig. 1
S1C	GATGACGGTATAGCAAGTGTG	21	Chol	
S2	GTTGGGAGGAGTGTGGAGCCAGCTACTCACTCAGATA GGGTA	42	-	
S3	TGGCTCCACACTCCTCCCAACCACACTTGCTATACCGTC ATC	42	-	
S4	TACCCTATCTGAGTGAGTAGC	21	Cy5	
S5	TACCCTATCTGAGTGAGTAGCTGGCTCCACACTCCTCCC AACCACACTTGCTATACCGTCATC	63	-	
S1α	GATGACGGTATAGCAAGTGTG	21	α-toco.	Fig. 2
S1DB	GATGACGGTATAGCAAGTGTG	21	DBCO	
S1DN	GATGACGGTATAGCAAGTGTG	21	DNP	
S6	CACACTTGCTATACCGTCATC	21	Cy5	
S7	CACACTTGCTATACCGTCATC	21	-	
S8a	AGTGAACAATTATATGGGCATGTTGGGAGGAGTGTGG AGCCA TTAACAGCTTGATGATTGACC	63	-	Fig. 3
S9a	GGTCAATCATCAAGCTGTTAATGGCTCCACACTCCTCCC AACATGCCCATATAATTGTTCACTCACACTTGCTATACC GTCATC	84	Cy5	
S8b	GTTGGGAGGAGTGTGGAGCCATTAACAGCTTGATGAT TGACC	42	-	
S9b	GGTCAATCATCAAGCTGTTAATGGCTCCACACTCCTCCC AACCACACTTGCTATACCGTCATCCACACTTGCTATACC GTCATC	84	Cy5	
S8c	TTAACAGCTTGATGATTGACC	21	-	
S9c	GGTCAATCATCAAGCTGTTAACACACTTGCTATACCGTC ATCCACACTTGCTATACCGTCATCCACACTTGCTATACC GTCATC	84	Cy5	
S9d	CACACTTGCTATACCGTCATCCACACTTGCTATACCGTC ATCCACACTTGCTATACCGTCATCCACACTTGCTATACC GTCATC	84	Cy5	
S10	CACACTTGCTATACCGTCATCCACACTTGCTATACCGTC ATCTTTTTCACACTTGCTATACCGTCATCCACACTTGCTA TACCGTCATC	89	Cy5	Fig. 4
S11a	CACTTGTTCTGAACGAGTTACTGTCTCGCTAAGAATATA CCACACTTGCTATACCGTCATC	61	-	
S11b	GTATATTCTTAGCGAGACAGAGGTCTGTGGCTGTACAA CCCACACTTGCTATACCGTCATC	61	-	
S11c	GGTTGTACAGCCACAGACCTGTATTGAGAAGTCTGTGA GGCACACTTGCTATACCGTCATC	61	-	

S11d	CCTCACAGACTTCTCAATACTAACTCGTTCAGAACAAGTGCACACTTGCTATACCGTCATC	61	-
S12a	CACTTGTTCTGAACGAGTTACTGTCTCGCTAAGAATATACTTTTTCACACTTGCTATACCGTCATC	66	-
S12b	GTATATTCTTAGCGAGACAGAGGTCTGTGGCTGTACAACCTTTTTCACACTTGCTATACCGTCATC	66	-
S12c	GGTTGTACAGCCACAGACCTGTATTGAGAAGTCTGTGAGGTTTTTCACACTTGCTATACCGTCATC	66	-
S12d	CCTCACAGACTTCTCAATACTAACTCGTTCAGAACAAGTGTTTTTCACACTTGCTATACCGTCATC	66	-
S13a	CACACTTGCTATACCGTCATCCACACTTGCTATACCGTCATCCACACTTGCTATACCGTCATCCACACTTGCTATACCGTCATC	84	-
S1DB2	GATGACGGTAGTGTG	15	DBCO
S14a	GAGCCATTAACAGCTTGATGATTG	24	-
S13b	CAATCATCAAGCTGTTAATGGCTCCACACTACCGTCATCCACACTACCGTCATCCACACTACCGTCATCCACACTACCGTCATC	84	-
S1DB3	GATGACGGTGCAAGTGTG	18	DBCO
S14b	GCTTGATGATTG	12	-
S13c	CAATCATCAAGCCACACTTGCACCGTCATCCACACTTGCACCGTCATCCACACTTGCACCGTCATCCACACTTGCACCGTCATC	84	-

Table S2. The transition temperature of applied lipids (details obtained from Avanti® Polar Lipids)

**Lipids**	**Chemical structure**	**Transition temperature (T_m)**
16:0 PC (DPPC)	1,2-dipalmitoyl-sn-glycero-3-phosphocholine 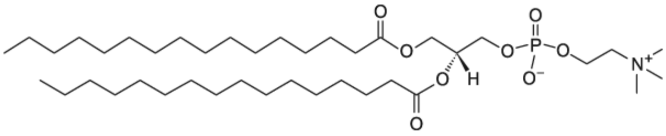	41 °C
14:0 PC (DMPC)	1,2-dimyristoyl-sn-glycero-3-phosphocholine 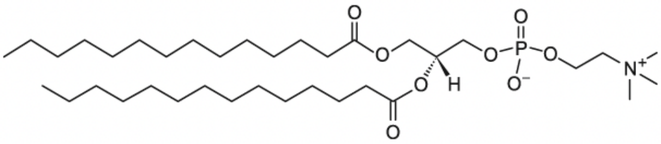	24 °C
16:0-18:1 PC (POPC)	1-palmitoyl-2-oleoyl-glycero-3-phosphocholine 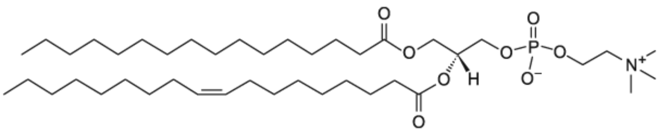	2 °C

Table S3. The applied DNA-lipid molar ratios in a variation of lipids patchs and DNA lengths described in the main figures. The lipid concentrations were calculated and standardized with PC phoshatidylcholine assay and the concentration of DNA is determined by the saturation curve in Figure S3. It is noted that a lower concentration of DNA is generally needed for DNA constrcuts with more negative charges.

DMPC [μM]	DNA[nM]	Length	DNA-lipid ratio	Saturation curve	Applied in
6.25	7.5	63 bp	~1:833	Fig S9a	Fig. 1
6.25	20	21 bp	~1:313	Fig S9b	Fig. 2c,d,e
6.25	15	84 bp	~1:417	Fig S9c	Fig. 3b,d
6.25	30	84 bp	~1:208	-	Fig. 3c
6.25	40	84 bp	~1:156	-	Fig. 4

Table S4. Summary of all the processed average change of zeta potential values ($|\Delta\bar{\zeta}|$). The data processing always involved the subtraction of the baseline (LUVs), and occasionally of the unmodified stand as the negative control. While STDEV stands for the stardard deviation (σ) of each group, N stands for the number of measurement for each replicate.

Group	N	X-axis	Avg |ΔZeta| ($|\Delta\bar{\zeta}|$)	STDEV (σ)	Used in
**Electrostatic**	1	15 °C	9.18	0.902	Fig. 1c
	2	15 °C	9.33		
	3	15 °C	6.97		
	4	15 °C	7.18		
	5	15 °C	8.34		
	6	15 °C	7.60		
	7	15 °C	8.13		
	8	15 °C	7.29		
	1	20 °C	6.17	0.920	
	2	20 °C	5.55		
	3	20 °C	8.00		
	4	20 °C	7.95		
	5	20 °C	7.74		
	6	20 °C	7.09		
	7	20 °C	6.25		
	8	20 °C	6.58		
	1	25 °C	2.29	0.512	
	2	25 °C	1.51		
	3	25 °C	2.61		
	4	25 °C	1.56		
	5	25 °C	2.17		
	6	25 °C	1.40		
	7	25 °C	2.26		

	8	25 °C	2.72		
	1	30 °C	1.52	0.495	
	2	30 °C	1.26		
	3	30 °C	2.00		
	4	30 °C	1.38		
	5	30 °C	0.99		
	6	30 °C	0.96		
	7	30 °C	0.43		
	8	30 °C	0.69		
	1	35 °C	1.23	0.424	
	2	35 °C	0.87		
	3	35 °C	1.09		
	4	35 °C	0.89		
	5	35 °C	0.14		
	6	35 °C	0.76		
	7	35 °C	0.22		
	8	35 °C	0.23		
**Hydrophobic**	1	15 °C	13.35	1.409	
	2	15 °C	13.06		
	3	15 °C	12.92		
	4	15 °C	12.52		
	5	15 °C	14.72		
	6	15 °C	14.48		
	7	15 °C	11.31		
	8	15 °C	10.62		
	1	20 °C	12.02	0.888	
	2	20 °C	11.26		
	3	20 °C	10.35		
	4	20 °C	10.74		
	5	20 °C	12.79		
	6	20 °C	12.75		
	7	20 °C	11.59		
	8	20 °C	12.14		
	1	25 °C	11.04	1.282	
	2	25 °C	11.63		
	3	25 °C	10.99		
	4	25 °C	12.47		
	5	25 °C	13.61		
	6	25 °C	14.70		
	7	25 °C	11.89		
	8	25 °C	12.65		

	1 30 °C	11.94	0.732	
	2 30 °C	12.75		
	3 30 °C	12.89		
	4 30 °C	13.33		
	5 30 °C	12.56		
	6 30 °C	13.13		
	7 30 °C	13.72		
	8 30 °C	14.34		
	1 35 °C	11.76	1.620	
	2 35 °C	10.86		
	3 35 °C	10.39		
	4 35 °C	10.44		
	5 35 °C	13.60		
	6 35 °C	14.92		
	7 35 °C	12.26		
	8 35 °C	13.03		
**Unmodified strand (Control)**	1 Ctrl	0.54	0.111	Fig. 2e
	2 Ctrl	0.73		
	3 Ctrl	0.73		
**α-tocopherol**	1 α-toco	15.53	0.751	
	2 α-toco	16.99		
	3 α-toco	15.94		
**Cholesterol**	1 Chol	14.96	0.898	
	2 Chol	15.08		
	3 Chol	16.57		
**DBCO**	1 DBCO	3.83	1.065	
	2 DBCO	4.50		
	3 DBCO	2.42		
**DNP**	1 DNP	1.81	0.560	
	2 DNP	2.61		
	3 DNP	1.53		
**4 α-tocopherol**	1 4-α-toco	11.98	0.565	Fig. 3b
	2 4-α-toco	12.87		
	3 4-α-toco	13.03		
**3-α-tocopherol**	1 3-α-toco	11.98	0.670	
	2 3-α-toco	12.87		
	3 3-α-toco	11.56		
**2-α-tocopherol**	1 2-α-toco	11.58	0.629	
	2 2-α-toco	12.79		
	3 2-α-toco	12.46		
**1-α-tocopherol**	1 1-α-toco	11.09	0.909	

	2 1-α-toco	12.79		
	3 1-α-toco	11.40		
**4-Cholesterol**	1 4-Chol	11.49	0.699	
	2 4-Chol	12.87		
	3 4-Chol	12.38		
**3-Cholesterol**	1 3-Chol	11.66	0.949	
	2 3-Chol	12.79		
	3 3-Chol	10.91		
**2-Cholesterol**	1 2-Chol	12.23	0.176	
	2 2-Chol	11.98		
	3 2-Chol	11.89		
**1-Cholesterol**	1 1-Chol	7.33	0.369	
	2 1-Chol	8.05		
	3 1-Chol	7.80		
**4-DBCO**	1 4-DBCO	1.33	0.454	
	2 4-DBCO	2.24		
	3 4-DBCO	1.82		
**3-DBCO**	1 3-DBCO	1.10	0.702	
	2 3-DBCO	1.86		
	3 3-DBCO	0.46		
**2-DBCO**	1 2-DBCO	-0.03	0.595	
	2 2-DBCO	0.68		
	3 2-DBCO	-0.51		
**1-DBCO**	1 1-DBCO	0.10	0.316	
	2 1-DBCO	0.67		
	3 1-DBCO	0.15		
**4-DNP**	1 4-DNP	0.41	0.493	
	2 4-DNP	1.24		
	3 4-DNP	0.37		
**3-DNP**	1 3-DNP	-0.11	0.487	
	2 3-DNP	0.84		
	3 3-DNP	0.17		
**2-DNP**	1 2-DNP	-0.74	0.512	
	2 2-DNP	0.22		
	3 2-DNP	-0.58		
**1-DNP**	1 1-DNP	-0.07	0.541	
	2 1-DNP	0.94		
	3 1-DNP	0.76		
**4-DBCO(2)**	1 4-DBCO	4.06	0.188	Fig. 3c
	2 4-DBCO	4.03		
	3 4-DBCO	4.37		

**3-DBCO(2)**	1	3-DBCO	4.10	0.181	
	2	3-DBCO	3.82		
	3	3-DBCO	3.76		
**2-DBCO(2)**	1	2-DBCO	2.21	0.215	
	2	2-DBCO	2.00		
	3	2-DBCO	2.43		
**1-DBCO(2)**	1	1-DBCO	1.81	0.242	
	2	1-DBCO	2.00		
	3	1-DBCO	1.52		
**4-DNP(2)**	1	4-DNP	1.58	0.231	
	2	4-DNP	1.84		
	3	4-DNP	1.38		
**3-DNP(2)**	1	3-DNP	1.60	0.125	
	2	3-DNP	1.83		
	3	3-DNP	1.63		
**2-DNP(2)**	1	2-DNP	1.45	0.055	
	2	2-DNP	1.44		
	3	2-DNP	1.54		
**1-DNP(2)**	1	1-DNP	1.19	0.055	
	2	1-DNP	1.20		
	3	1-DNP	1.29		
**Linear**	1	DBCO-L	8.00	0.732	Fig. 4d
	2	DBCO-L	8.35		
	3	DBCO-L	6.94		
**Flexible Linear**	1	DBCO-FL	5.00	0.882	
	2	DBCO-FL	6.68		
	3	DBCO-FL	5.38		
**Tetrapod**	1	DBCO-TP	9.65	0.904	
	2	DBCO-TP	11.43		
	3	DBCO-TP	10.83		
**Flexible Tetrapod**	1	DBCO-FTP	7.71	1.060	
	2	DBCO-FTP	6.40		
	3	DBCO-FTP	5.62		
**Chol-parrallel**	1	Chol-P	21.00	0.353	Fig. 4f
	2	Chol-P	20.36		
	3	Chol-P	20.94		
**Unmodified control**	1	Ctrl	2.06	0.337	
	2	Ctrl	2.29		
	3	Ctrl	2.72		
**DBCO-parrallel**	1	DBCO-P	6.60	1.235	

	2	DBCO-P	7.92		
	3	DBCO-P	5.46		
**DBCO-anti-parallel**	1	DBCO-AP	3.30	0.513	
	2	DBCO-AP	4.32		
	3	DBCO-AP	3.89		
**DBCO-orthogonal**	1	DBCO-O	2.95	0.618	
	2	DBCO-O	2.62		
	3	DBCO-O	1.75		

References

(1) Zadeh, J. N.; Steenberg, C. D.; Bois, J. S.; Wolfe, B. R.; Pierce, M. B.; Khan, A. R.; Dirks, R. M.; Pierce, N. A. NUPACK: Analysis and Design of Nucleic Acid Systems. *J. Comput. Chem.* **2011**, *32* (1), 170–173.

(2) Fornace, M. E.; Huang, J.; Newman, C. T.; Porubsky, N. J.; Pierce, M. B.; Pierce, N. A. NUPACK: Analysis and Design of Nucleic Acid Structures, Devices, and Systems. **2022**.

(3) Angelova, M.; Soléau, S.; Méléard, P.; Faucon, F.; Bothorel, P. Preparation of Giant Vesicles by External AC Electric Fields. Kinetics and Applications. *Trends Colloid Interface Sci. VI* **1992**, 127–131.

(4) Morzy, D.; Rubio-Sánchez, R.; Joshi, H.; Aksimentiev, A.; Di Michele, L.; Keyser, U. F. Cations Regulate Membrane Attachment and Functionality of DNA Nanostructures. *J. Am. Chem. Soc.* **2021**, *143* (19), 7358–7367. https://doi.org/10.1021/jacs.1c00166.

(5) Morzy, D.; Tekin, C.; Caroprese, V.; Rubio-Sánchez, R.; Di Michele, L.; Bastings, M. M. C. Interplay of the Mechanical and Structural Properties of DNA Nanostructures Determines Their Electrostatic Interactions with Lipid Membranes. *Nanoscale* **2023**, *15* (6), 2849–2859. https://doi.org/10.1039/D2NR05368C.

(6) Douglas, S. M.; Marblestone, A. H.; Teerapittayanon, S.; Vazquez, A.; Church, G. M.; Shih, W. M. Rapid Prototyping of 3D DNA-Origami Shapes with caDNAno. *Nucleic Acids Res.* **2009**, *37* (15), 5001–5006. https://doi.org/10.1093/nar/gkp436.

(7) Lee, J. Y.; Lee, J. G.; Yun, G.; Lee, C.; Kim, Y.-J.; Kim, K. S.; Kim, T. H.; Kim, D.-N. Rapid Computational Analysis of DNA Origami Assemblies at Near-Atomic Resolution. *ACS Nano* **2021**, *15* (1), 1002–1015. https://doi.org/10.1021/acsnano.0c07717.

(8) Lee, J. Y.; Kim, M.; Lee, C.; Kim, D.-N. Characterizing and Harnessing the Mechanical Properties of Short Single-Stranded DNA in Structured Assemblies. *ACS Nano* **2021**, *15* (12), 20430–20441. https://doi.org/10.1021/acsnano.1c08861.

(9) Poppleton, E.; Romero, R.; Mallya, A.; Rovigatti, L.; Šulc, P. OxDNA.Org: A Public Webserver for Coarse-Grained Simulations of DNA and RNA Nanostructures. *Nucleic Acids Res.* **2021**, *49* (W1), W491–W498. https://doi.org/10.1093/nar/gkab324.

(10) Rovigatti, L.; Šulc, P.; Reguly, I. Z.; Romano, F. A Comparison between Parallelization Approaches in Molecular Dynamics Simulations on GPUs. *J. Comput. Chem.* **2015**, *36* (1), 1–8. https://doi.org/10.1002/jcc.23763.

(11) Snodin, B. E. K.; Randisi, F.; Mosayebi, M.; Šulc, P.; Schreck, J. S.; Romano, F.; Ouldridge, T. E.; Tsukanov, R.; Nir, E.; Louis, A. A.; Doye, J. P. K. Introducing Improved Structural Properties and Salt Dependence into a Coarse-Grained Model of DNA. *J. Chem. Phys.* **2015**, *142* (23), 234901. https://doi.org/10.1063/1.4921957.

(12) Humphrey, W.; Dalke, A.; Schulten, K. VMD: Visual Molecular Dynamics. *J. Mol. Graph.* **1996**, *14* (1), 33–38. https://doi.org/10.1016/0263-7855(96)00018-5.
